# Synthesis of Novel VO(II)-Perimidine Complexes: Spectral, Computational, and Antitumor Studies

**DOI:** 10.1155/2018/7176040

**Published:** 2018-09-06

**Authors:** Gamil A. Al-Hazmi, Khlood S. Abou-Melha, Nashwa M. El-Metwaly, Kamel A. Saleh

**Affiliations:** ^1^Chemistry Department, Faculty of Science, King Khalid University, P.O. Box 9004, Abha, Saudi Arabia; ^2^Chemistry Department, Faculty of Applied Sciences, Taiz University, P.O. Box 82, Taiz, Yemen; ^3^Chemistry Department, College of Applied Sciences, Umm Al-Qura University, Makkah, Saudi Arabia; ^4^Chemistry Department, Faculty of Science, Mansoura University, Mansoura, Egypt; ^5^Biology Department, Faculty of Science, King Khalid University, P.O. Box 9004, Abha, Saudi Arabia

## Abstract

A series of perimidine derivatives (L^1–5^) were prepared and characterized by IR, ^1^H·NMR, mass spectroscopy, UV-Vis, XRD, thermal, and SEM analysis. Five VO(II) complexes were synthesized and investigated by most previous tools besides the theoretical usage. A neutral tetradentate mode of bonding is the general approach for all binding ligands towards bi-vanadyl atoms. A square-pyramidal is the configuration proposed for all complexes. XRD analysis introduces the nanocrystalline nature of the ligand while the amorphous appearance of its metal ion complexes. The rocky shape is the observable surface morphology from SEM images. Thermal analysis verifies the presence of water of crystallization with all coordination spheres. The optimization process was accomplished using the Gaussian 09 software by different methods. The most stable configurations were extracted and displayed. Essential parameters were computed based on frontier energy gaps with all compounds. QSAR parameters were also obtained to give another side of view about the biological approach with the priority of the L^3^ ligand. Applying AutoDockTools 4.2 program over all perimidine derivatives introduces efficiency against 4c3p protein of breast cancer. Antitumor activity was screened for all compounds by a comparative view over breast, colon, and liver carcinoma cell lines. IC_50_ values represent promising efficiency of the L^4^-VO(II) complex against breast, colon, and liver carcinoma cell lines. The binding efficiency of ligands towards CT-DNA was tested. Binding constant (*K*_b_) values are in agreement with the electron-drawing character of the p-substituent which offers high *K*_b_ values. Also, variable Hammett's relations were drawn.

## 1. Introduction

Vanadium was widely used as a therapeutic agent in the late eighteenth century, treating a variety of ailments including anemia, tuberculosis, rheumatism, and diabetes [1, 2]. Vanadium compounds exhibit various biological and physiological effects in the human body. Vanadium compounds have been extensively studied for their diverse biological activities such as antitumor, antibacterial, and insulin-enhancing effects and potential capabilities as DNA structural probes [3, 4]. The coordination chemistry of oxovanadium is highly ligand dependent and more important in biological systems [5] as well as catalytic systems [6, 7]. Due to the d1 configuration, vanadium(IV) ionic species are easily identified by EPR spectroscopy. Due to less toxicity [8, 9], the Schiff base complexes of the vanadyl ion are topic of many research reports [10, 11]. In Europe, vanadium is often used as a natural treatment for diabetes. Vanadium has been found in human studies to imitate the effects of insulin in our bodies. This ability may be useful for some of those with diabetes, a natural method to help lower blood sugar, take less insulin, or in some instances stop taking insulin altogether [12, 13]. It is noticeable that complexation of vanadium with organic ligands minimizes unfavorable effects of its inorganic salts such as vanadyl sulfate while even maintains its potential benefits [14]. Furthermore, mimicking the biological activities in natural systems can be achieved by vanadium complexes which contain oxygen and nitrogen donor ligands; so identification of the structure of these complexes is regarded important [15–17]. Bioinorganic chemistry is a fast developing field of modern chemistry that uses Schiff bases and their transition metal complexes for a variety of applications, e.g., in biological, medical, and environmental sciences. This work is interested in preparation of a series of perimidine derivatives by various substituents. New vanadyl complexes will be prepared and well characterized by using different techniques. CT-DNA binding will be tested along the organic series. Theoretical implementation will be accomplished over all prepared compounds by different standard programs. Antitumor activity will be scanned over all new prepared compounds for comparison.

## 2. Experimental Work

### 2.1. Chemicals Used

Chemicals essential for preparation of perimidine derivatives such as 1,8-diaminonaphthalene, ethylbenzoyl acetate, 4-methoxyaniline, aniline, 4-chloroaniline, 3-chloroaniline, 4-nitroaniline, NaNO_2_, NaOH, HCl, and dioxane were purchased from Fluka and used without previous treatments. Also, VOSO_4_·xH_2_O salt used for the complexation process was commercially available from Sigma-Aldrich. All handled solvents were from Merck and used without previous purification.

### 2.2. Synthesis

#### 2.2.1. Synthesis of Compounds **3a–e** (Perimidine Derivatives)

**Scheme 1:**
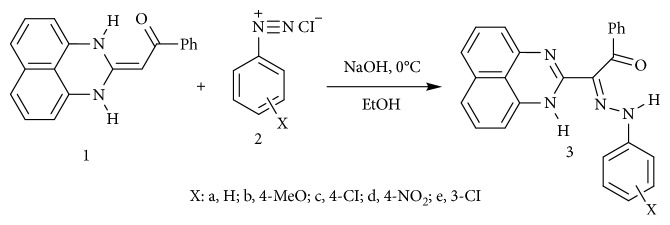
Synthesis of perimidine compounds **3a–e**.

**Figure 1:**
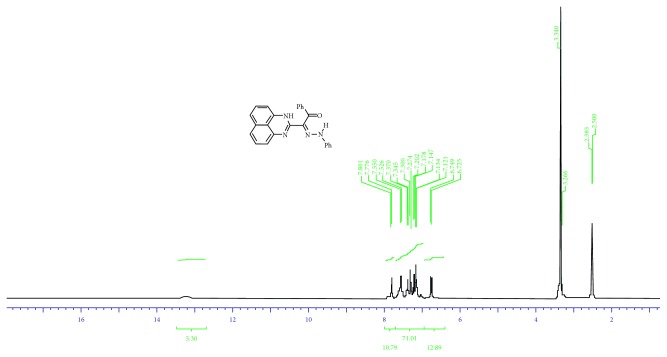
^1^H·NMR of L^1^ ligand (as example).

**Figure 2:**
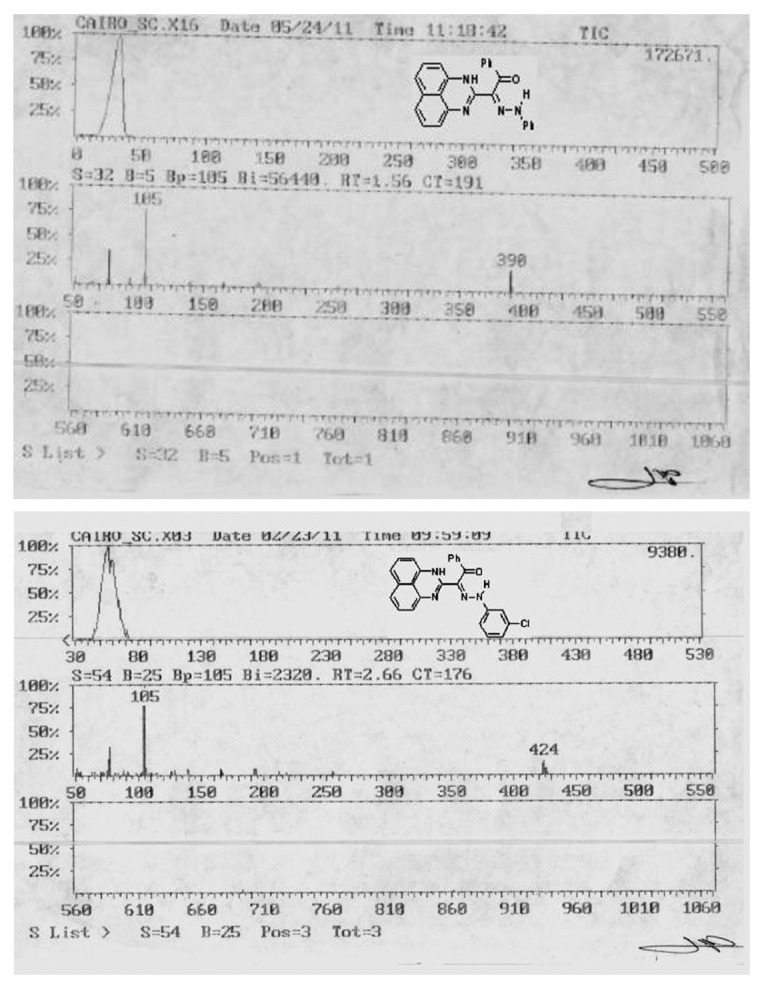
Mass spectra of L^1^ and L^4^ ligands.

**Figure 3:**
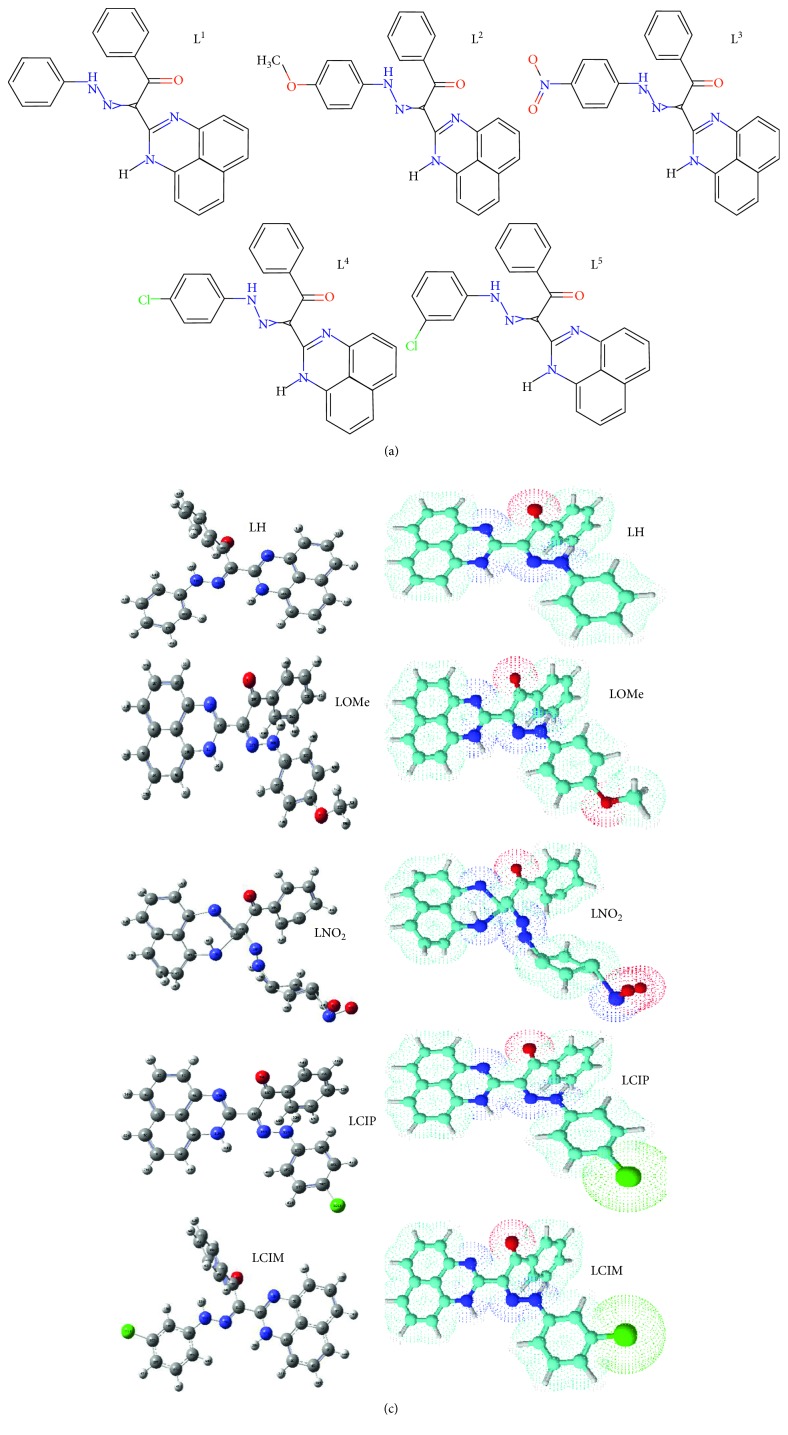
(a) Structures of perimidine ligands (L^1–5^). (b) Optimized structures of five perimidine ligands.

Ligands **3a–e** were synthesized as reported in the literature [18] from coupling reaction of compound 1 (2.5 mmol) in ethanol (20 mL) with the appropriate arenediazonium chloride 2 in the presence of sodium hydroxide (2.5 mmol) in the ice bath at 0–5°C. The whole mixture was then left in a refrigerator overnight. The precipitated solid was filtered off, washed with water, and finally crystallized from dioxane/EtOH to give the respective hydrazones **3a–e** (Scheme 1). ^1^H·NMR and mass spectra are displayed in Figures 1, 2, S1, and S2. The analysis is matching completely with that reported in the literature [16]. The structural forms of new perimidine compounds are displayed in Figures 3(a) and 3(b).

1. *2-[N-phenyl-2-oxo-2-phenylethanehydrazonoyl]-1H-perimidine (* ***3a*** *) (L* ^*1*^). IR *υ*: 3402, 3194 (2NH), and 1616 (CO) cm^−1^. ^1^H·NMR (DMSO-d_6_) d: 6.72–7.80 (m, 16H, Ar–H) and 13.62 (s, 2H, 2NH). MS *m*/*z* (%): 390 (M, 22), 285 (2), 166 (5), 140 (7), 127 (1), 105 (100), 93 (8), and 77 (35). Anal. calcd. for C_25_H_18_N_4_O (390.42).
2. *2-[N-(4-methoxyphenyl)-2-oxo-2-phenylethanehydrazonoyl]-1H-perimidine (* ***3b*** *) (L* ^*2*^). IR *υ*: 3333, 3167 (2NH), and 1680 (CO) cm^−1^. ^1^H·NMR (DMSO-d_6_) *δ*: 3.58 (s, 3H, OCH_3_), 7.59–7.97 (m, 15H, ArH), and 12.20 (br s, 2H, 2NH). MS *m*/*z* (%): 420 (M, 5), 419 (9), 193 (12), 166 (12), 126 (14), 107(17), 105 (100), 92 (31), and 77 (75). Anal. calcd. for C_26_H_20_N_4_O_2_ (420.47).
3. *2-[N-(4-chlorophenyl)-2-oxo-2-phenylethanehydrazonoyl]-1H-perimidine (* ***3c*** *) (L* ^*3*^). IR *υ*: 3422, 3206 (2NH), and 1612 (CO) cm^−1^. ^1^H·NMR (DMSO-d_6_) d: 6.73–7.77 (m, 15H, Ar–H) and 13.27 (s, 2H, 2NH). MS *m*/*z* (%): 426 (M 2, 5), 425 (M 1, 6), 424 (M, 14), 140 (4), 127 (5), 111 (2), 105 (100), and 77 (37). Anal. calcd. for C_25_H_17_ClN_4_O (435.44).
4. *2-[N-(4-*nitrophenyl*)-2-oxo-2-phenylethanehydrazonoyl]-1H-perimidine (* ***3d*** *) (L* ^*4*^). IR *υ*: 3356, 3198 (2NH), and 1670 (CO) cm^−1^. ^1^H·NMR (DMSO-d_6_) d: 6.64–8.21 (m, 15H, Ar–H) and 12.60 (s, 2H, 2NH). MS *m*/*z* (%): 435 (M, 8), 238 (34), 138 (13), 167 (11), 106 (45), 105 (58), 93 (100), 77 (44), and 66 (76). Anal. calcd. for C_25_H_17_N_5_O_3_ (424.88).
5. *2-[N-(3-*chlorophenyl*)-2-oxo-2-phenylethanehydrazonoyl]-1H-perimidine (* ***3e*** *) (L* ^*5*^). IR *υ*: 3229, 3167 (2NH), and 1622 (CO) cm^−1^. ^1^H·NMR (DMSO-d_6_) d: 6.70–7.91 (m, 15H, Ar–H) and 13.02 (s, 2H, 2NH). MS *m*/*z* (%): 426 (M2, 7), 425 (M1, 9), 424 (M, 16), 194 (7), 166 (7), 140 (5), 127 (4), 105 (100), 111 (4), and 77 (36). Anal. calcd. for C_25_H_17_ClN_4_O (424.88).

#### 2.2.2. Synthesis of VO(II) Complexes

New VO(II) complex series was synthesized by using variable derivatives from perimidine ligands. Equimolar (3 mmol) values were used from the perimidine ligand and dissolved fully in dioxane; after that, it was mixed with VOSO_4_·xH_2_O which dissolves in the dioxane/H_2_O mixture. The weighted molar ratio value from vanadyl salt was calculated attributing to its anhydrous weight. After ≈5 h reflux, 0.5 g sodium acetate was added after dissolving in a little amount of bi-distilled water to precipitate the complexes. Each precipitate was separated out on hot, filtered off, washed several times with ethanol and diethyl ether, and finally dried in a vacuum desiccator.

### 2.3. DNA Binding Study

The binding attitudes of perimidine derivatives towards calf thymus DNA (CT-DNA) will be studied by using the spectroscopy method. CT-DNA (50 mg) was dissolved by stirring overnight in double deionized water (pH = 7.0) and must be kept at 4°C. Bi-distilled water was used to prepare the buffer (5.0 mM tris(hydroxymethyl)-aminomethane and 50 mM NaCl, pH = 7.2). Tris-HCl buffer was prepared in deionized water. DNA buffering solution gave absorbance ratio at 260/280 nm by 1.8–1.9, and this indicates the absence of protein from DNA [19, 20]. Applying the UV-Vis technique, the DNA concentration was determined (5.10 × 10^−4^ M) using its known molar absorptivity coefficient value (6600 M^−1^·cm^−1^ at 260 nm). At room temperature, 200–900 nm is the wavelength range used, and in 1 cm quartz cuvette, a fixed concentration (2.0 × 10^−5^ M in dioxane) from each ligand was utilized. A scanning process was done after adding CT-DNA by a gradual way from 0.00 to ≈2.18 × 0^−4^ mol·L^−1^. The same DNA amount added to the ligand solution was added also to the reference cell to delete the absorbance of free DNA. A significant binding constant (*K*_b_) for interaction between ligands towards CT-DNA was determined by using the following equation: [DNA]/(*є*_a_ − *є*_f_) = [DNA]/(*є*_b _− *є*_f_) + 1/*K*_b_ (*є*_a_ − *є*_f_) [21], where [DNA] is the concentration of CT-DNA in base pairs, *є*_a_ is the extinction coefficient observed for A/[compound] at the used DNA concentration, and *є*_f_ is the extinction coefficient for each free compound (HL^1−5^) in the solution. Moreover, *є*_b_ is the extinction coefficient of the compound when fully bond to DNA. In plots of [DNA]/(*є*_a_ − *є*_f_) vs. [DNA], *K*_b_ is given by the following ratio: slope/intercept.

### 2.4. Antitumor Influence

The evaluation of cytotoxicity of candidate anticancer drugs will be performed using the most effective, available SRB method. All molecules and their derivatives will be tested for their toxicity on different cancer cell lines. In an attempt to evaluate the impact, the samples were prepared with different concentrations: 0.01, 0.1, 1, 10, and 100 *µ*g/ml, respectively. The cells were cultured in the mixture of samples and media (RPMI-FBS + samples) for 72 h; after that, cytotoxicity impact was evaluated compared to the response of doxorubicin as a positive control.

The cytotoxic effect of the composites and ligands will be tested against different cancer cell lines (HepG2, MCF-7, and HCT116) as donor cancer cell lines by means of the SRB cytotoxicity test. To avoid the contamination, the RPMI media of the cells were supplemented with 100 *µ*g/ml streptomycin and 100 units/ml penicillin with 10% FBS and incubated at a 5% CO_2_ incubator. Growing cells were collected using the trypsin enzyme and then counted using the cell counter in order to distribute equally the number of cells to each well of 69-well plates. The cells will incubate under sterile conditions with different concentrations of both ligands and composites for 72 hours, and subsequently, treated cells and untreated cells and the positive control were fixed with 10% TCA (trichloroacetic acid) and kept at 4°C for 1 h. After washing few times, fixed and washed cells were stained with 0.4% SRB stain solution for ten minutes, and subsequently, the cells were washed with 1% glacial acetic acid. To dissolve SRB-stained cells, Tris-HCl was used. To detect the density of remaining colors, a plate reader will be used at 540 nm wavelength. In order to determine the IC_50_ value, statistical analysis was accomplished through SigmaPlot version 14.0. The advantage of prepared compounds as potential drugs against different cancer cells was investigated.

### 2.5. Physical Techniques

#### 2.5.1. Elemental Analysis

The element contents (carbon, hydrogen, and nitrogen) were determined at the Micro-Analytical Unit of Cairo University. Vanadium, sulfate, and chloride contents were evaluated by known standard methods [22] through complexometric and precipitation methods.

#### 2.5.2. Conductivity Measurements

Applying the Jenway 4010 conductivity meter, the molar conductivity of freshly prepared 1.0 × 10^−3^ mol/cm^3^ in DMSO solutions was estimated.

#### 2.5.3. X-Ray Diffraction and SEM

X-ray diffraction manners were recorded on the Rigaku diffractometer using Cu/K*α* radiation. Scanning electron microscopy (SEM) images were obtained by using Joel JSM-6390 equipment.

#### 2.5.4. IR, ^1^H·NMR, and ^13^CNMR Spectra

IR spectra were obtained using the JASCO FT/IR-4100 spectrophotometer from 400 to 4000 cm^−1^ in the KBr disc, while ^1^H·NMR spectra were recorded in deuterated dimethyl sulfoxide using the Varian Gemini 300 NMR spectrometer.

#### 2.5.5. Mass spectra

Mass spectra were recorded on GCMS-QP1000 EX (Shimadzu) and GCMS 5988-A.

#### 2.5.6. ESR Analysis

ESR spectra of VO(II)-powdered complexes were obtained on the Bruker EMX spectrometer working in the X-band (9.60 GHz) with 100 kHz modulation frequency. The microwave power was set at 1 mW, and modulation amplitude was set at 4 Gauss. The low field signal was obtained after 4 scans with a 10-fold increase in the receiver gain. A powder spectrum was obtained in a 2 mm quartz capillary at ordinary temperature.

#### 2.5.7. UV-Vis Spectra and Magnetic Measurements

Electronic spectra for all compounds were recorded using the UV_2_ Unicam UV/Vis spectrophotometer in the DMSO solvent. Magnetic susceptibility values for VO(II) complexes were conducted by the Johnson Matthey magnetic susceptibility balance at room temperature.

#### 2.5.8. Thermal Analysis

The Shimadzu thermogravimetric analyzer (20–900°C) at 10°C·min^−1^ heating rate under nitrogen was used for thermal analysis. Theoretical treatments (modeling and docking) were accomplished by known standard programs.

#### 2.5.9. Antitumor Activity

Antitumor activity was conducted at the Regional Center for Mycology and Biotechnology.

### 2.6. Computational

#### 2.6.1. DFT/Hartree–Fock Study

Implementing the Gaussian 09 software [23], the structural optimization process was accomplished over pyrimidine ligands and their VO(II) complexes in the gas phase. Two known methods were found as the most suitable one for the optimization process. The output files were visualized by the GaussView program [24]. According to the numbering scheme, DFT parameters were extracted using frontier energy gaps (*E*_HOMO_ and *E*_LUMO_) for all investigated compounds. Moreover, other significant computations were taken from log files as oscillator strength, excitation energy, charges assigned for coordinating atoms, and some bond lengths.

#### 2.6.2. QSAR Computation

New perimidine compounds were treated for the optimization process to give the best structural forms. HyperChem (v8.1) software is the tool used for such a purpose. The preoptimization process was executed by molecular mechanics force field (MM^+^) accompanied by semiempirical AM1 for the soft adjustment procedure. This process was accomplished without fixing any parameter till the equilibrium state for geometric structures. A system for minimizing energy was employed the Polak–Ribiere conjugated gradient algorithm. The QSAR process leads to computing essential parameters including the partition coefficient (log *P*). Log *P* value is considered the essential indicator used to predict the biological activity for optimized compounds [25].

#### 2.6.3. Docking Computation

Applying AutoDockTools 4.2 by using Gasteiger partial charges which added over the elements of pyrimidine ligands, the simulation procedure was executed to give a view on the biological behavior of compounds. Rotatable bonds were cleared, and nonpolar hydrogen atoms were conjoined. Interaction occurred between inhibitors (ligands) and protein receptors (4c3p, 3bch, and 4zdr) for breast, colon, and liver cancer proteins. The docking process was accomplished after addition of fundamental hydrogen atoms, Kollman united atom-type charges, and salvation parameters [26]. Affinity (grid) maps of ×× Å grid points and 0.375 Å spacing were generated applying the AutoGrid program [27]. Van der Waals forces and electrostatic terms were obtained. This is done by applying autodock parameter set-dependent and distance-dependent dielectric functions, respectively. The docking process was executed using the Solis and Wets local search method and Lamarckian genetic algorithm (LGA) [28]. Initial position, orientation, and torsions of the inhibitor molecule were set indiscriminately. All rotatable torsions were expelled during the docking process. Each experiment is the mean value of 10 different runs that are set close after the maximum of 250000 energy assessments. 150 is the used population size. During the process, the translational step of 0.2 Å, quaternion, and torsion steps of 5 were applied.

## 3. Results and Discussion

### 3.1. Physical Properties

Essential analytical and physical data for ligands and their VO(II) complexes are summarized in Table 1. All investigated complexes are nonhygroscopic in nature, having high melting point (>300°C). The elemental analysis proposes 1 : 2 (HL : M) molar ratio as the general formula for all complexes. All complexes are completely soluble in DMSO or DMF solvents. The conductivity measured is 5.66–14.22 Ω^−1^·cm^2^·mol^−1^. Such conductivity values are attributed to the nonconducting character of tested complexes [29]. This coincides with sulfate anion which favors covalent attachment with metal ions inside the coordination sphere.

### 3.2. Comparative IR Study

The assignments of all characteristic bands for five perimidine ligands and their VO(II) complexes are summarized in Table 2. *ν*(NH), *δ*(NH), *ν*(C=N), and *ν*(C=O) are the significant functional bands for coordinating groups which appear in narrow regions observed in all derivatives. This may refer to the far effect of the aromatic substituent on bond movement inside such groups. A comparative study of ligands and their VO(II) complexes reveals the following observations: (1) lower-shifted appearance of former bands is considered a strong evidence for contribution of C=O, NH, and C=N groups in coordination towards two central atoms. (2) New bands appeared at 1368–1434 and 1140–1179 cm^−1^ assigned for *ν*_as_(SO_4_) and *ν*_s_(SO_4_), respectively, through bidentate attachment [30]. (3) Other bands appearing at ≈760 and ≈690 cm^−1^ are attributed to *δ*_r_(H_2_O) and *δ*_w_(H_2_O), respectively, for crystal water molecules. (4) *ν*(M-L) bands appeared at the low wavenumber region belonging to M-O and M-N bonds. These spectral observations suggest a tetradentate mod of coordination towards two vanadyl atoms. Also, the band observed at 966–1074 cm^−1^ range assigns for *ν*(V = O), significantly pointing to the square-pyramidal configuration [31].

### 3.3. Electronic Spectra and Magnetic Measurements

**Figure 4:**
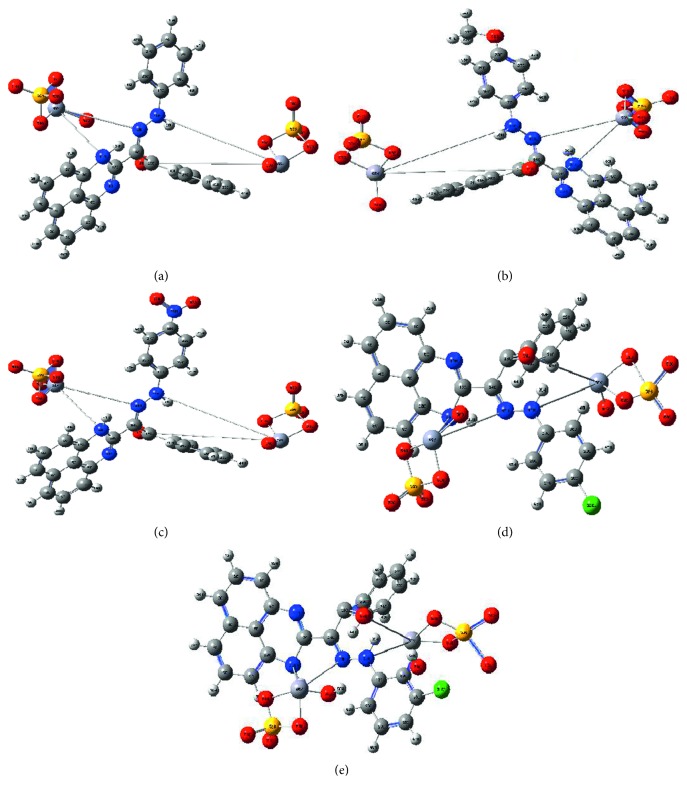
Geometry optimization of VO(II)-perimidine complexes (a–e, respectively).

Electronic transition bands and magnetic moment values are aggregated in Table 3. UV-Vis spectra were recorded qualitatively in the DMSO solvent to gain smoothly absorption curves. Intraligand transition bands appearing at 31,250–38,168, 25,974–30,769, and 17,544–19,157 cm^−1^ are attributed to *n* ⟶ σ^*∗*^, *π* ⟶ *π* ^*∗*^, and *n* ⟶ *π* ^*∗*^ transitions, respectively, inside variable groups [32]. A structural condensed conjugation of chromophores leads to appearance of deep colors for all perimidine ligands. This is accompanied with the appearance of the *n* ⟶ *π* ^*∗*^ band in the middle of the visible region. VO(II) complex spectra display intraligand transitions suffer shift due to coordination. The appearance of charge transfer bands attributes to O ⟶ V and N ⟶ V transitions. Also, new significant d-d transition bands were observed at ≈15,300 and 12,800 cm^−1^ assigned for ^2^B_2_g ⟶ ^2^B_1_g (*E*_2_, *υ*_2_) and ^2^B_1_g ⟶ ^2^Eg (*E*_1_, *υ*_1_), respectively. These bands are attributed to transition inside the square-pyramidal configuration (Figure 4). Reduced magnetic moment values (*μ*_eff_ = 1.65–1.68 BM) recorded for all complexes support the proposal of binuclear complexes [33].

### 3.4. ESR Spectra

**Figure 5:**
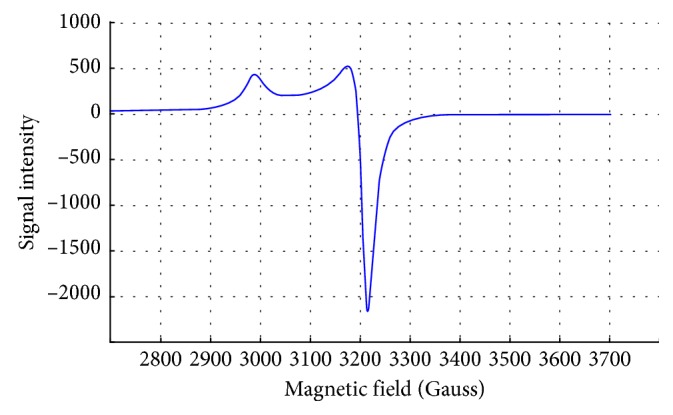
ESR spectrum of L^1^ + VO(II)complex.

ESR spectra (Figure 5, for example) of VO(II) solid complexes were obtained and investigated to verify the structural forms of them. All spectra demonstrated an eight-line pattern, which attributes to the analogous and vertical ingredients g-tensors and hyperfine (hf) A-tensors. Spin Hamiltonian parameters and molecular orbital values were calculated and are represented in Table 4. The analogous and vertical ingredients are well resolved. Nitrogen super-hyperfine splitting is not observed, which points to the presence of single electron in the *d*_xy_ orbital. The pattern suggests that *g* and *A* are axially symmetric in nature. The factors *A* and *g* appear to be in covenant with the values commonly known for vanadyl complex in the square-pyramidal geometry. *G* factor, which is expressed by *G* = (*g*_//__ _− 2.0023)/(*g*_⊥ _− 2.0023) = 4, measures the exchange interaction between metal centers. In agreement with Hathaway [34, 35], *G* > 4 shows negligible exchange interaction, while *G* < 4 is the vice versa. An observable reduction of the values calculated (1.71–2.79) proposes strong interaction inside binuclear complexes [36, 37]. This interaction affects the magnetic moment value of complexes which suffers observable reduction. The tendency of *A*_11_ to decrease with increasing *g*_11_ is an index for tetrahedral distortion (*f*=*g*_//_/*A*_//_) [38–40]. The molecular orbital coefficients *α* ^2^ and *β* ^2^ are calculated by

(1) $$\begin{array}{c} \beta^{2}=\frac{7}{6}\left(-\frac{A_{11}}{P}+\frac{A_{⊥}}{P}+g_{11}-\frac{5}{14}g_{⊥}-\frac{9}{14}g_{\text{e}}\right),\\ \alpha^{2}=\frac{2.0023-\Delta g}{8\beta^{2}\lambda },\;\text{where }\Delta g=\left(g_{⊥}-g_{\left|\right|}\right)\times 10^{-3}. \end{array}$$

The hyperfine conjunction disciplinarians were calculated by taking A_//_ and A_⊥_ as negative, which gave positive values of *β* ^2^ and *α* ^2^. The calculated *α* ^2^ and *β* ^2^ values introduce the highly ionic character of in-plane *σ*- and *π*-bonding. The electronic transition spectra display two significant bands at ≈15,300 and 12,800 cm^−1^ assigned for ^2^B_2_g ⟶ ^2^B_1_g (*E*_2_, *υ*_2_) and ^2^B_1_g ⟶ ^2^Eg (*E*_1_, *υ*_1_), respectively. Assume pure d-orbitals by using first- and second-order perturbation theories. The parameters attributing to transition energy are called the spin Hamiltonian parameters and calculated by the following expression: *g*_⊥_ = *g*_e_ − (2*λ*/*E*_2_), where *g*_e_ is the free-electron *g* value (2.0023). Using *E*_2_ value, the spin-orbital coupling constant (*λ*) was evaluated (138.18). A value for *λ* of 250 cm^−1^ is reported [41] for free V^+4^ ion. The high reduction in the magnitude of *λ* for the double-bonded oxovanadium complex (V=O)^+2^ is attributed to substantial *π*-bonding. However, the value falls inside the logical borders announced. The orbital reduction factors, namely, *K*_//_ and *K*_⊥_, are also calculated using ^2^ *K*_//_ = (*g*_//_ − 2.00277) *E*/8 *λ* and ^2^ *K*_⊥_ = (*g*_⊥_ − 2.00277) *E*/2 *λ*. For pure *σ*-bonding, *K*_//_ ≈ *K*_⊥_ ≈ −0.77, while ^2^ *K*_// _> ^2^ *K*_⊥_ signifies in-plane *σ*-bonding, with ^2^ *K*_⊥ _< ^2^ *K*_//_ accounting for out-of-plane *π*-bonding [42].

#### 3.4.1. Calculation of Dipole Term (*p*)

Dipolar term values can be determined by

(2) $$\begin{array}{c} p=\frac{7\left(A_{11}-A_{⊥}\right)}{6+\left(3/2\right)\left(\lambda /E_{1}\right)}. \end{array}$$

If *A*_11_ is taken to be negative and *A*_⊥_ positive, the value of *p* will be more than 270 *G*, which is far from the expected value. Thus, the signs of both *A*_11_ and *A*_⊥_ are used as negative and are indicated in the form of the isotropic hf constant (*A*_o_). McGarvey theoretically accomplished the *p* value as +136 *G* for vanadyl complexes which does not deviate much from the expected value.

#### 3.4.2. Calculation of MO Coefficients and Bonding Parameters

The *g* values observed are different from the electronic value (2.0023). This assigns to spin-orbit interaction of the *d*_xy_ ground state level. The isotropic and anisotropic *g* and *A* parameters were calculated using the following equations: *A*_o_ = (*A*_//_ + 2*A*_⊥_)/3 and *g*_o_ = (*g*_//_ + 2*g*_⊥_)/3. Taking *A*_11_ and *A*_⊥_ to be negative values, the *K* expression is *K* = −(*A*_o_/*p*) − (*g*_e__ _− *g*_o_).

Thus, *K* (Fermi contact term) can be determined. The Fermi contact term, *k*, is a sense of polarization exerted by the uneven apportionment of d-electron density on the inner core s-electron.

### 3.5. X-Ray Diffraction

**Figure 6:**
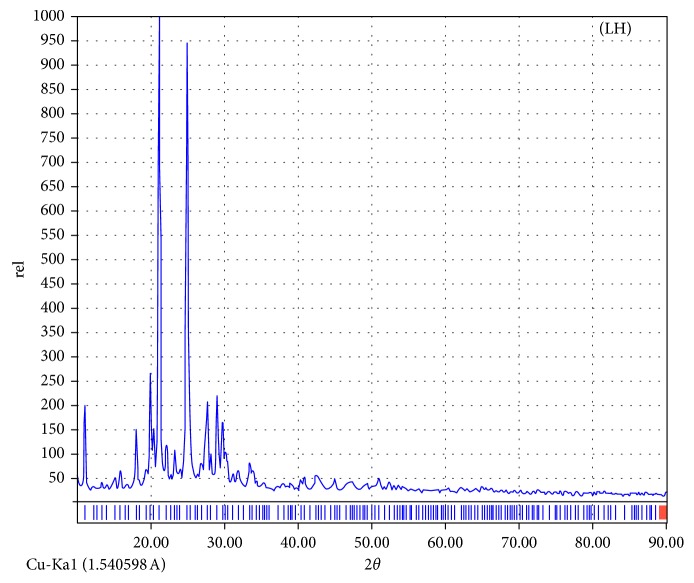
X-ray diffraction pattern of high crystalline ligand.

X-ray diffraction patterns were executed over 10° < 2*θ* < 90° range (Figures 6 and S3) for ligands under study. This technique gives a considerable view about dynamics of the crystal lattice in solid compounds. Using standard methods, a comparative study of patterns with reactants reflects the purity of isolated compounds [43]. Also, significant parameters related to crystalline compounds can be calculated using the high-intense peak (full width at half maximum (FWHM)). The crystallinity appearing with the LH ligand reflects the isolation of a strictly known irregular crystallite, while the amorphous appearance of others reflects the indiscriminate orientation of atoms inside the 3D space. 2*θ* (21.18), *d* spacing (4.1910), FWHM (0.2454), relative intensity (%) (857), and particle size (6.003 *Ε*) were calculated for LH compounds. The crystallite size was computed by utilizing the Debye–Scherrer equation: *β* = 0.94 *λ*/(S cos *θ*), where *S* is the crystallite size, *θ* is the diffraction angle, *β* is FWHM, and Cu/K*α* (*λ*) = 1.5406 Å. The d-spacing between inner crystal planes was extracted from the Bragg equation: *nλ* = 2dsin(*θ*) at *n* = 1. The size calculated falls in the nanometer range (nm) which expects a widespread application especially for the biological field. Also, crystal strain (*ε*, 5.027) was calculated by *β* = (*λ*/*S*  cos  *θ*) − *ε*  tan  *θ*, while dislocation density (*δ*, 0.0277) was computed by *δ* = 1/*S* ^2^ [44]. The dislocation density and strain are the aspects for network dislocation in compounds. The lower values of them indicate high quality of compounds. The SEM tool is used to give a clear view about the habit and surface morphology of all studied compounds (Figure S4). The images of paramagnetic compounds are not strictly resolved because an insufficient electron beam can meet the surface to provide well resolution. Subsequently, the determination of particle size in an accurate way is strongly absent. It was known about this study that the crystals were grown up from just a single one to several accumulated distributables with particle sizes starting with few nanometers to many hundreds. The formation of extended crystals over a rocky shape may happen by two nucleation processes: by distribution and by piling up of layers which are grown. It was pointed to that if the rate of growth along the *C*-axis is fast and a great number of grown nuclei are active across the axis in comparison with vertical to the *C*-axis, the crystals will be extended over patches [45]. The attitude displayed on different crystals may be due to the growth along the strongest bond through anisotropy included in crystal structures. When the amount exceeds to a certain limit, the result is evolution of plates and rock shapes. It is credible to assume that the environmental conditions change the nature and shape of the morphology. Moreover, the rock and plates shaping compounds may have excellent activity towards different applications due to their broad surface area [46]. The homogeneous morphology observed indicates the obtained strict-defined crystals are free from metal ions on the external surface.

### 3.6. Thermal Study

The degradation behaviors of all perimidine compounds and their VO(II) complexes were tested. The proposed degradation insights corresponding to all decomposition stages are tabulated (Table 5). The treated perimidine compounds start their successive decomposition at low temperature (≈60°C) in three stages. A sequenced complete degradation of the organic compound was recorded with or without carbon atoms residue. VO(II)-perimidine complexes display an observable thermal stability for the organic compounds coordinated. The degradation stages varied in between three and four stages. The first degradation process starts at 40–80°C temperature range which starts with the removal of water molecules and is followed by decomposition of the coordinating ligand. Variable residue was proposed with the complexes degradation process but all agree with the presence of biatomic metals. An acceptable conformity between calculated and found weight losses percentage may reflect the exact determination of stage borders.

### 3.7. DNA Binding

**Figure 7:**
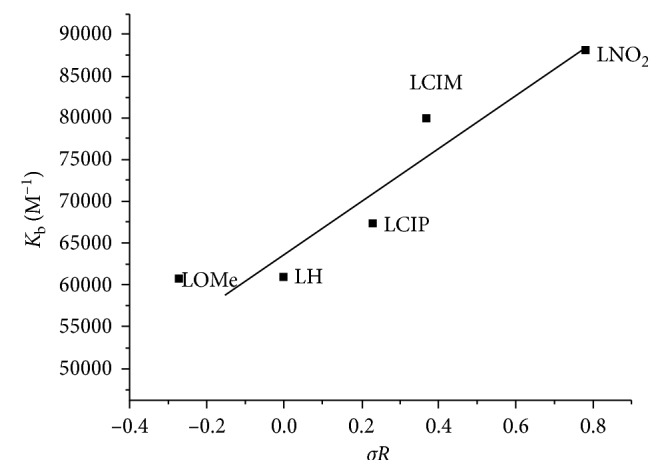
Hammett's relation between the effect of p-substituent (*σR*) and intrinsic binding constants (*K*_b_) of the ligands.

Appling the spectrophotometric titration method, the binding mod of perimidine derivatives towards CT-DNA was investigated. Electronic absorption of freshly prepared solutions was obtained at 25°C over 200–800 nm range, with a reference solution for each concentration. Scanned solutions include fixed ligand concentration (2 × 10^−5^ M) with a regular increase of DNA added. The effective binding constant for the interaction of the organic derivatives with DNA was obtained based on observable changes in absorption at 418, 420, 420, 385, and 410 nm for LH, LOMe, LNO_2_, L^4^, and L^5^, respectively. A regular increase of DNA amount added to the ligand solution leads to the bathochromic effect for the significant ligand band assigned for transition inside interacting groups. This band is minimized gradually as appeared clearly with the aggregated spectra for each derivative. This minimization is followed by appearance of the slightly shifted peak (1-2 nm) from the free ligand peak, which assigns for the binding complex and suffers a gradual increase in absorbance. This is considered as a sufficient indicator of coupled DNA helix stabilization, after the interaction process. Such an investigation suggests the coupling for binding sites through electrostatic attraction or occluded in major and minor grooves inside DNA. Also, the bathochromic effect can be investigated and explained based on two bases: broad surface area of perimidine molecules and the presence of planar aromatic chromophore, which facilitate well binding towards CT-DNA. This groove binding leads to structural reorganization of CT-DNA. This requires a partial disassembling or deterioration of double helix at the exterior phosphate, which leads to formation of cavity suitable for entering compounds [47]. The bathochromic feature observed is directly proportional to electron withdrawing character for substituents and their position. The binding constants (*K*_b_) for the five derivatives were calculated by known spectral relationships [19] for L^1^, L^2^, L^3^, L^4^, and L^5^ as 6.10 × 10^4^, 6.07 × 10^4^, 6.75 × 10^4^, 7.99 × 10^4^, and 8.80 × 10^4^ M^−1^. According to Hammett's constants (*σ*_R_), essential correlation against intrinsic constants will be conducted (Figure 7), and the relation verifies the direct relation in between [48].

### 3.8. Computational

#### 3.8.1. DFT/Hartree–Fock Study

**Figure 8:**
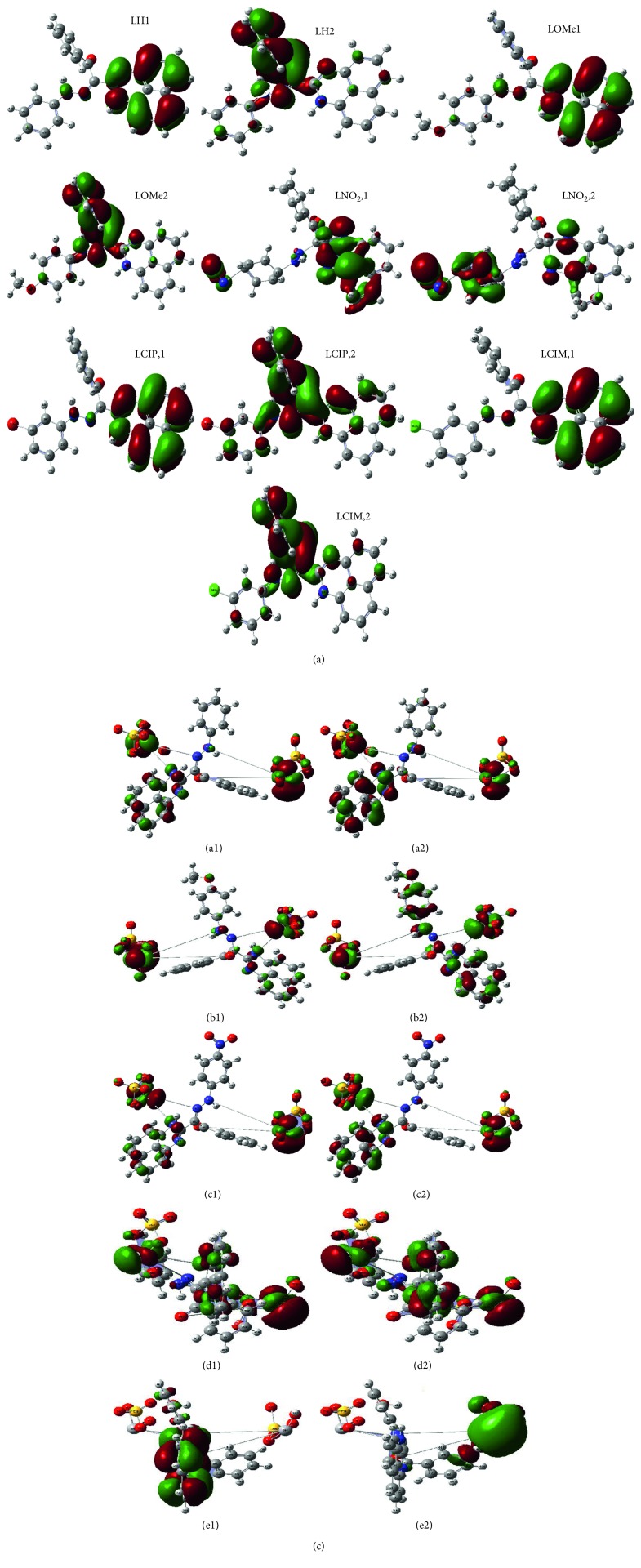
(a) Frontier molecular orbitals of HOMO(1)and LUMO(2) pictures of perimidine ligands. (b) Frontier molecular orbitals of HOMO(1) and LUMO(2) pictures of VO(II)-perimidine complexes (A–E, respectively).

Applying the Gaussian 09 software, the optimization process was executed over all new compounds till reaching the best configuration. A known standard method was used for this purpose. Essential parameters will be extracted from the energy levels of frontiers (HOMO and LUMO). The energy gap between *E*_HOMO_ and *E*_LUMO_ will give an excellent view about the character of the tested compound. The biological behavior and the ligational mode are most significant features concluded. The frontier images of perimidine ligands and their VO(II) complexes are shown in Figures 8(a) and 8(b), respectively. HOMO-level images display the concentration over the perimidine ring which includes two donor centers, while the LUMO-level images display the concentration over the other side in molecules including the other two coordination sites. This view introduces a good electron relocation between donor atoms which smoothens the donation of coordinating centers. On the other side, the two levels in VO(II) complexes represent the concentration around the two central atoms mainly. This may offer the good role of VO atoms in the application feature interested in this research. This may happen through the smooth charge transfer process that includes the complexes. Electronegativity (*χ*), chemical potential (*μ*), global hardness (*η*), global softness (*S*), global electrophilicity index (*ω*), and absolute softness (ϭ) were calculated by using known standard equations [49, 50]. Toxicity and reactivity of compounds can be clarified by using the electrophilicity index (*ω*) value. This index gives a clear insight about the expected biological attitude of tested perimidine compounds in comparison with their VO(II) complexes and, also, measures the firmness of the compound which takes an extra negative charge from the environment. Also, the firmness and reactivity of compounds can be tested from two opposite indexes (*η* and ϭ) [42, 51].

*(1) Some Quantum Parameters*. Some important quantum parameters are calculated for all treated compounds attributing to frontier energy gaps and are displayed in Table 6. The computed results of ligands introduce the following notices: (i) the degree of converged softness recorded for perimidines offers their compatible flexibility towards the coordination. (ii) Electrophilicity index (*χ*) and electronic chemical potential index (*μ*) have two different signs. This is evidence for the ability of compounds to acquire electrons from the surrounding by the following order: L^4^ > L^3^ > L^5^ > L^1^ > L^2^ ligands. This arrangement agrees by an excellent way with the priority of electron withdrawing substituents (Cl and NO_2_) in para position which facilitates the compound electron affinity.

**Figure 9:**
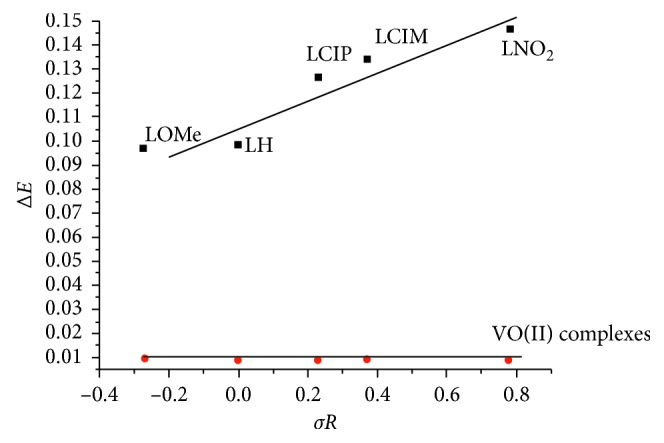
Hammett's relation between the effect of p-substituent (*σR*) and energy gaps (*δE*) of ligands and their VO(II) complexes.

Whenever, the extracted data assigning for VO(II) complexes introduce the following observations: (i) frontier energy gaps are completely minimized from original perimidines leading to red shift inside electronic transitions. Such a behavior may clarify the effect of metal atoms (vanadyl) in stabilizing the compounds. This reduction is preferable in biological attitude of compounds [52]. (ii) The absolute softness values in complexes were enhanced than the ligand values which predicated their high biological activity. From calculated energy gaps, Hammett's relation displays a significant effect of the p-substituent on *δE* values of ligands or their complexes by two reverse features (Figure 9).

*(2) Some Log File Parameters*. Essential log file data are summarized and presented in Table 7. The allowed data are varied in between the free ligands and their complexes, due to the difference in methods used for the treatment. A suitable method used for the organic ligand appeared unsuitable for its VO(II) complex. A comparative investigation introduced the following notices: (i) a general reduction in the charges computed for coordinating atoms (O^19^, N^11^, N^15^, and N^16^). This is due to their participation in coordination with VO(II) atoms. (ii) The computed bond lengths appearing with four perimidine derivatives are comparable with each other except for the L^3^ ligand. This displays the inductive effect of the p-substituent (nitro group) on the elongation of bond lengths attributing to the affected function groups. (iii) Oscillator strength values (range 0–1) of complexes are commonly minimized than those of their corresponding ligands. This may indicate the effect of the metal atom (vanadyl) in facilitating the absorption or reemission of electromagnetic radiations inside complex molecules [53]. The values are close to zero and not 1; this may suggest low excitation energy values needed for electronic transitions. (iv) Also, an increase in dipole moment values of complexes over ligands indicates high polarity of covalent bonds surrounding two central atoms except for the L^2^ complex. This may refer to the significant difference between all substituents from the methoxy group which has electron-donating feature in opposite with the others [54].

#### 3.8.2. QSAR Calculations

Using the HyperChem (v8.1) program, essential QSAR parameters are calculated and tabulated (Table 8). This computation gives a clear view about some statistics belonging to coordinating agents. Log *P* value is an indication for the biological feature of the tested compound by a reverse relation [55]. The values are arranged by the following order: L^1^ (2.53) > L^4^ = L^5^ (2.31) > L^2^ (1.53) > L^3^ (−1.64). Partition coefficient (log *P*) values introduce a distinguish biological activity may appear with the L^3^ ligand.

#### 3.8.3. Docking Computation

**Figure 10:**
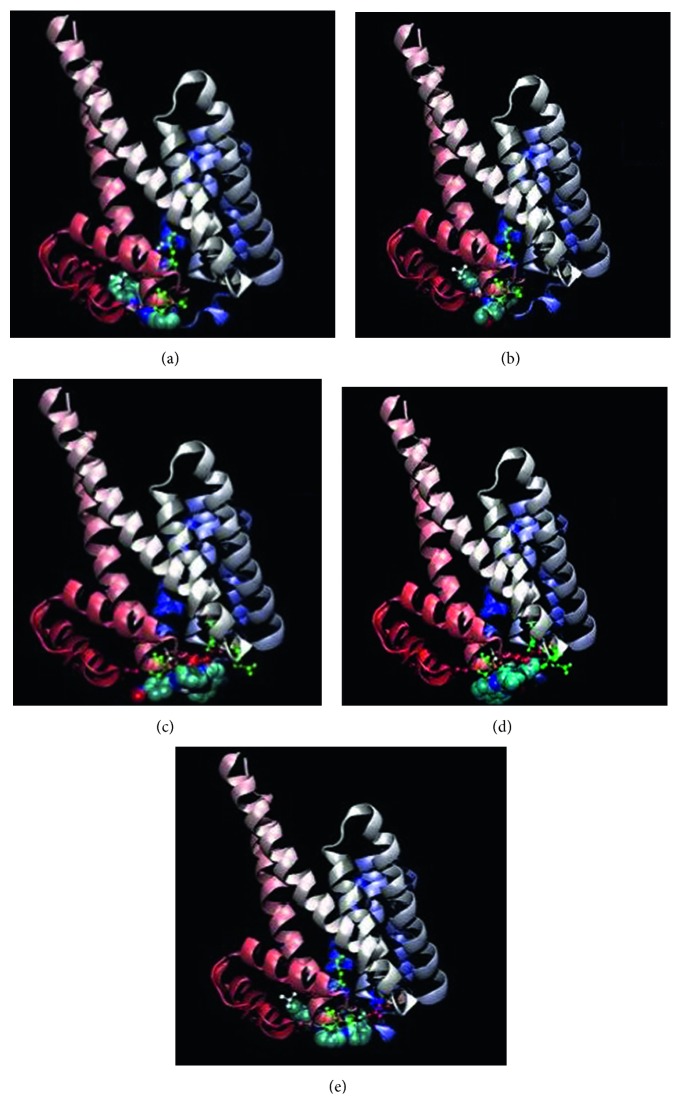
Interacting protein-inhibitor complexes (a) L^1^, (b) L^2^, (c) L^3^, (d) L^4^, and (e) L^5^ with 4zdr receptor (a–e, respectively).

**Figure 11:**
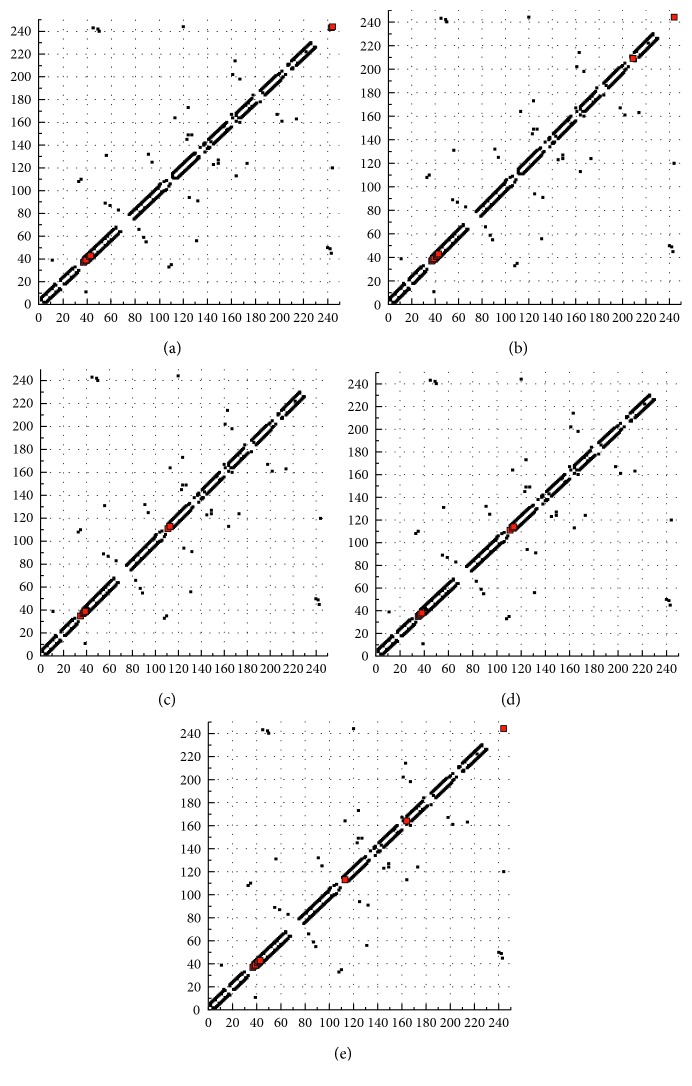
Interacting complexes hp plot for (a) L^1^, (b) L^2^, (c) L^3^, (d) L^4^, and (e) L^5^ with 4zdr receptor (a–e, respectively).

Simulation technique is a new revolution process served in different applications. Drug design is a complicated process that needs significant facilities to establish a view about the expected efficiency of proposed drugs. In last decades, the docking computation process between the proposed drug (inhibitor) and the infected cell proteins is the concern in drug industrial research. AutoDockTools 4.2 software was used for this approach. 4c3p, 3bch, and 4zdr are the BDP files for breast, colon, and liver cancer cell proteins which are used for the docking process with five perimidine derivatives. The extracted energies over PDB files (a format using the Gaussian 09 software) are presented in Table 9. Scanning for the energy values introduces the following observations: (i) there is no interaction observed with the five inhibitors towards 3bch colon cancer protein. (ii) The degree of interaction towards breast colon protein (4c3p) is arranged as L^5^ > L^1^ > L^2^ > L^4^, while the arrangement towards 4zdr (liver cancer protein) is as L^5^ > L^1^ > L^4^ > L^3^ > L^2^. This result displays the priority of L^5^ and L^1^ ligands in the inhibition process towards breast and liver carcinoma cell lines through a strong interaction (Figures 10 and S5) [55]. Dissociation constant (p*K*_a_) calculated is considered the biopharmaceutical measure of drug-likeness compounds. This constant helps in understanding the ionic form of the drug along the pH range. High p*K*_a_ values (>10) reflect their ionization which facilitates their diffusion across the cell membrane to give a well inhibition process. Also, highly reduced energies were recorded for 4c3p and 4zdr receptors. Positive sign of electrostatic energy recorded clarifies high stability of interacting complexes. HP plots (Figures 11 and S6) as well as 2D plots (Figure S7) display prolonged H-bonding appearing with L^5^ and LH ligands. This verifies the degree of interaction proposed on extracted energies. Also, high surface area recorded with breast or liver cancer protein complexes introduces a good degree of H-interaction. And hp, 2D, and surface area data verify the absence of interaction recorded with colon cancer protein.

### 3.9. Antitumor Efficiency

**Figure 12:**
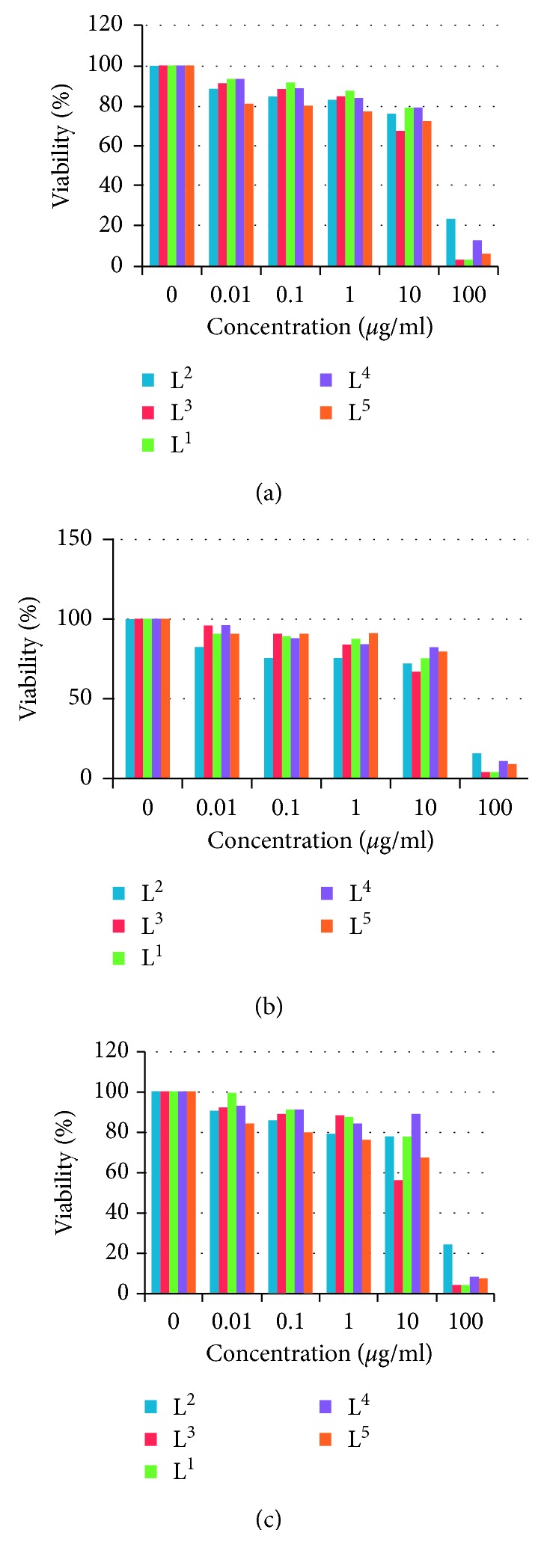
Dose response curves of perimidine ligands against MCF-7 (a), HCT116 (b), and HepG2 (c) cancer cells.

The results obtained by screening all prepared compounds for comparison confirmed that the complexes exhibit more cytotoxicity against HepG2, MCF-7, and HCT116 *in vitro*. The IC_50_ values are displayed in Table 10 and in Figures 12 and S8. Cells were treated with various concentrations of compounds and incubated for 48 h [56]. Cytotoxicity is considered as a good anticancer parameter if the influence induced apoptotic pathways inside the cell. Apoptotic may be detected by many parameters like the activation of caspase family, DNA fragmentation, or morphology of the cell. The sample L^4^ + VO(II) was the best impacted complex on liver, breast, and colon cancer cell lines with IC_50_ values of 1.66, 3.42, and 1.27, respectively, while its relative ligand (L^4^) impact was moderate at all cancer types. Hence, these results showed that the present study's effort to improve and enhance the effect of new complexes has achieved a clear, acceptable, and respectable success because the effect was enhanced for 15, 7, and 22 times, respectively, compared to the ligand with clear signs that it is going to be very close to the positive control. On the contrary, unfortunately, the same results were not detected in other complexes; they went in the contrary way: instead, they increased the impact they decreased it dramatically. What our results tell us clearly is that neither VO(II) nor ligands alone can act as an anticancer candidate drug, while only one complex can present that effect. So, the mechanism of action is not related to the ligand or to VO(II) itself, as far as related to the complex itself.

## 4. Conclusion

This paper presents new VO(II) complexes derived from a series of perimidine ligands. This study focuses on the effect of substituents on the chemistry and applicability of complexes. All the new compounds were well characterized by all possible tools. The complexes were found in a nanoscale comfortably. The different theoretical implementations gave a view about the biological feature of the investigated compounds in a comparative way. The docking process displays the high interaction of organic derivatives against breast cancer, while the experimental investigation displays the priority of the L^4^-VO(II) complex against all carcinomas tested. The binding efficiency of ligands towards CT-DNA was tested. Binding constant (*K*_b_) values are in agreement with the electron-drawing character of the p-substituent which displayed high *K*_b_ values.

## Figures and Tables

**Table 1:** Significant analytical and physical data of perimidine compounds and their VO(II) complexes.

Compounds (formula weight) (calcd./found)	Color	Elemental analysis (%) calcd. (found)				
		C	H	N	SO_4_/Cl	V
(**1**) (C_25_H_18_N_4_O) (L^1^) (390.44/390.42)	Dark brown	76.91 (76.90)	4.65 (4.66)	14.35 (14.35)	—	—
(**2**) [(VO)_2_ (SO_4_)_2_ (L^1^)]H_2_O (734.46)	Dark brown	40.88 (40.88)	2.74 (2.73)	7.63 (7.65)	26.16 (26.16)	13.87 (13.88)
(**3**) (C_26_H_20_N_4_O_2_) (L^2^) (420.47/420.44)	Dark brown	74.27 (74.27)	4.79 (4.79)	13.32 (13.31)	—	—
(**4**) [(VO)_2_ (SO_4_)_2_ (L^2^)]2H_2_O (782.51)	Dark green	39.91 (39.90)	3.09 (3.10)	7.16 (7.17)	24.55 (24.54)	13.02 (13.03)
(**5**) (C_25_H_17_N_5_O_3_) (L^3^) (435.44/435.42)	Dark brown	68.96 (68.95)	3.93 (3.93)	16.08 (16.09)	—	—
(**6**) [(VO)_2_ (SO_4_)_2_ (L^3^)]H_2_O (779.46)	Dark green	38.52 (38.52)	2.46 (2.46)	8.99 (8.97)	24.65 (24.66)	13.07 (13.05)
(**7**) (C_25_H_17_N_4_OCl) (L^4^) (424.88/424.86)	Dark brown	70.67 (70.66)	4.03 (4.02)	13.19 (13.18)	8.34 (8.35)	—
(**8**) [(VO)_2_ (SO_4_)_2_ (L^4^)]H_2_O (768.90)	Dark green	39.05 (39.05)	2.49 (2.48)	7.29 (7.28)	24.99 (25.02)/4.61 (4.63)	13.25 (13.26)
(**9**) (C_25_H_17_N_4_OCl) (L^5^) (424.88/424.86)	Dark brown	70.67 (70.68)	4.03 (4.05)	13.19 (13.18)	8.34 (8.35)	—
(**10**) [(VO)_2_ (SO_4_)_2_ (L^5^)]3H_2_O (804.93)	Dark brown	37.30 (37.31)	2.88 (2.88)	6.96 (6.95)	23.87 (23.88)/4.40 (4.41)	12.66 (12.67)

**Table 2:** Significant IR spectral bands (cm^−1^) of perimidine compounds and their VO(II) complexes.

Compounds	*ν*_NH,_ *ν*_OH_	*δ*_NH_	*υ*_C=O_	*ν*_C=N_	*ν*_as(SO4)_	*ν*_s(SO4)_	*δ*r(H_2_O), *δ*w(H_2_O)	*ν*_V=O_	*ν*_M-O_	*ν*_M-N_
(**1**) (C_25_H_18_N_4_O) (L^1^)	3155	1473	1618	1518	—	—	—	—	—	—
(**2**) [(VO)_2_ (SO_4_)_2_ (L^1^)]H_2_O	3110, 3350	1470	1596	1514	1420	1142	765, 670	966	588	476
(**3**) (C_26_H_20_N_4_O_2_) (L^2^)	3177	1470	1597	1508	—-	—	—	—	—	—
(**4**) [(VO)_2_ (SO_4_)_2_ (L^2^)]2H_2_O	3100, 3372	1447	1592	1502	1411	1146	765, 697	1074	572	515
(**5**) (C_25_H_17_N_5_O_3_) (L^3^)	3150	1473	1620	1538	—	—	—	—	—	—
(**6**) [(VO)_2_ (SO_4_)_2_ (L^3^)]H_2_O	3105, 3382	1447	1616	1517	1411	1179	743, 697	966	600	508
(**7**) (C_25_H_17_N_4_OCl) (L^4^)	3160	1474	1614	1518	—	—	—	—	—	—
(**8**) [(VO)_2_ (SO_4_)_2_ (L^4^)]H_2_O	3100, 3420	1471	1624	1510	1434	1150	755, 637	985	610	550
(**9**) (C_25_H_17_N_4_OCl) (L^5^)	3150	1473	1620	1518	—	—	—	—	—	—
(**10**) [(VO)_2_ (SO_4_)_2_ (L^5^)]3H_2_O	3054, 3384	1446	1616	1512	1368	1140	754, 689	1074	589	508

**Table 3:** Electronic transitions of perimidine compounds and their VO(II) complexes.

Compounds	*μ*_eff_ (BM)	d-d transition bands (cm^−1^)	Intraligand and charge transfer (cm^−1^)
(**1**) (C_25_H_18_N_4_O) (L^1^)	—	—	31,746; 26,316; 23,923; 18,868
(**2**) [(VO)_2_ (SO_4_)_2_ (L^1^)]H_2_O	1.66	15,290; 12800	35,714; 29,412; 25,641; 24,272; 17,857
(**3**) (C_26_H_20_N_4_O_2_) (L^2^)	—	—	38,168; 28,249; 23,810; 19,048
(**4**) [(VO)_2_ (SO_4_)_2_ (L^2^)]2H_2_O	1.68	15,393; 12750	37,037; 30,769; 26,316; 23,256; 17,544
(**5**) (C_25_H_17_N_5_O_3_) (L^3^)	—	—	36,364; 30,303; 26,316; 23,810; 19,157
(**6**) [(VO)_2_ (SO_4_)_2_ (L^3^)]H_2_O	1.66	15,873; 12830	37,037; 28,571; 26,667; 18,182
(**7**) (C_25_H_17_N_4_OCl) (L^4^)	—	—	31,746; 25,974; 18,587
(**8**) [(VO)_2_ (SO_4_)_2_ (L^4^)]H_2_O	1.67	15,385; 12,800	35,714; 30,303; 25,974; 24,390; 17,857
(**9**) (C_25_H_17_N_4_OCl) (L^5^)	—	—	31,250; 26,316; 24,390; 18,182
(**10**) [(VO)_2_ (SO_4_)_2_ (L^5^)]3H_2_O	1.65	15,873; 12780	35,714; 30,769; 25,000; 23,256; 18,868

**Table 4:** Spin Hamiltonian parameters of all VO(II) complexes (A and *p* x10^−4^).

Complex	*g*_//_	*g*_⊥_	*g*_o_	*A*_11_	*F*	*A*_⊥_	*A*_o_	*G*	*p*	*k*	^2^ *K*_//_	^2^ *K*_⊥_	*α* ^2^	*β* ^2^
(**1**)	1.93	1.96	1.95	167	115.57	66	99.67	1.71	117.52	0.796	−0.843	−1.981	1.959	0.9357
(**2**)	1.94	1.97	1.96	170	114.12	71	104.00	1.93	115.19	0.861	−0.724	−1.512	1.490	0.9435
(**3**)	1.92	1.96	1.95	168	114.28	69	105.67	1.95	115.19	0.865	−0.961	−1.985	1.964	0.9243
(**4**)	1.94	1.98	1.97	171	113.45	73	105.67	2.79	114.00	0.895	−0.727	−1.055	1.033	0.9395
(**5**)	1.93	1.97	1.96	171	112.86	72	105.00	2.24	115.19	0.869	−0.841	−1.515	1.494	0.9318

**Table 5:** Estimated TG data of perimidine compounds and all VO(II) complexes.

Compound	Steps	Temp. range (°C)	Decomposed	Weight loss; calcd. (found %)
L^1^	1^st^	45.1–120.5	-[C_6_H_6 _+ N_2_]	27.18 (27.16)
	2^nd^	122.2–410.1	-[C_6_H_5 _+ CO]	26.92 (26.95)
	3^rd^	410.3–670.2	-[C_8_H_7_N_2_]	33.59 (33.54)
	Residue		4C	12.31 (12.35)
[(VO)_2_ (SO_4_)_2_ (L^1^)]H_2_O	1^st^	80.3–120.3	-[H_2_O + SO_4_]	15.53 (15.55)
	2^nd^	120.6–391.7	-[SO_4 _+ C_6_H_6 _+ N_2_]	27.53 (27.55)
	3^rd^	391.9–798.8	-[C_19_H_12_N_2_]	36.53 (36.50)
	Residue		V_2_O_3_	20.41 (20.40)
L^2^	1^st^	65.6–156.1	-[C_6_H_5_OCH_3_]	25.72 (25.71)
	2^nd^	156.6–299.9	-[C_6_H_5 _+ CO + N_2_]	31.66 (31.69)
	3^rd^	301.0–663.2	-[C_9_H_7_N_2_]	34.05 (33.89)
	Residue		3C	8.57 (8.71)
[(VO)_2_ (SO_4_)_2_ (L^2^)]2H_2_O	1^st^	42.1–135.1	-[2H_2_O + 2SO_4_]	29.16 (29.16)
	2^nd^	136.1–270.1	-[C_6_H_5_OCH_3 _+ N_2_]	17.40 (17.29)
	3^rd^	271.0–485.4	-[C_6_H_5_]	9.85 (9.94)
	4^th^	485.6–797.9	-[C_13_H_7_N_2_]	24.43 (24.45)
	Residue		V_2_O_3_	19.15 (19.16)
L^3^	1^st^	63.66–230.51	-[C_6_H_6_ + NO_2_]	28.50 (27.90)
	2^nd^	231.21–410.11	-[C_7_H_5 _+ CO + N_2_]	33.33 (33.31)
	3^rd^	410.52–650.64	-[C_11_H_6_N_2_]	38.16 (38.79)
[(VO)_2_ (SO_4_)_2_ (L^3^)]H_2_O	1^st^	79.2–140.6	-[H_2_O + SO_4_]	14.63 (14.62)
	2^nd^	141.9–278.9	-[SO_4_ + C_6_H_5 _+ CO]	25.81 (25.78)
	3^rd^	279.1–479.5	-[NO_2 _+ C_6_H_6 _+ N_2_]	19.52 (19.53)
	4^th^	480.11–798.8	-[C_12_H_6_N_2_]	22.86 (22.90)
	Residue		V_2_O_2_	17.18 (17.17)
L^4^	1^st^	64.65–145.46	-[C_6_H_5_Cl + CO + N_2_]	39.68 (39.68)
	2^nd^	145.68–326.78	-[C_6_H_5 _+ N_2_]	24.74 (24.75)
	3^rd^	330.12–680.23	-[C_12_H_7_]	35.58 (35.57)
[(VO)_2_ (SO_4_)_2_ (L^4^)]H_2_O	1^st^	69.1–256.1	-[H_2_O + C_6_H_5_Cl + SO_4_]	29.48 (29.65)
	2^nd^	256.9–484.1	-[SO_4_ + CON_2 _+ C_6_H_5_]	29.80 (29.81)
	3^rd^	484.6–789.4	-[C_10_H_7_N_2_]	20.18 (19.98)
	Residue		V_2_O_2 _+ 2C	20.54 (20.56)
L^5^	1^st^	62.3–169.6	-[C_6_H_5 _+ N_2_]	24.74 (24.71)
	2^nd^	160.1–371.9	-[C_6_H_5_Cl + CO + N_2_]	39.68 (39.59)
	3^rd^	372.6–666.8	-[C_9_H_7_]	27.10 (27.19)
	Residue		3C	8.48 (8.51)
[(VO)_2_ (SO_4_)_2_ (L^5^)]3H_2_O	1^st^	42.1–266.3	-[3H_2_O + C_6_H_5_Cl]	20.70 (20.71)
	2^nd^	266.38–482.5	-[2SO_4_ + CON_2 _+ C_6_H_5_]	40.41 (40.61)
	3^rd^	482.9–793.5	-[C_8_H_7_N_2_]	16.29 (16.36)
	Residue		V_2_O_2 _+ 4C	22.60 (22.32)

**Table 6:** Energy parameters (eV) using the DFT/B3LYP method of optimized structures.

Compound	*E*_H_	*E*_L_	(*E*_H_ − *E*_L_)	*E*_L_ − *E*_H_	*x*	*µ*	*η*	*S* (eV^−1^)	*ω*	ϭ
L^1^	−0.17417	−0.07574	−0.0984	0.09843	0.124955	−0.12496	0.049215	0.024608	0.158628	20.31900843
L^1 ^+ VO(II)	−0.20433	−0.19545	−0.0089	0.00888	0.19989	−0.19989	0.00444	0.00222	4.499551	225.2252252
L^2^	−0.17142	−0.07426	−0.0972	0.09716	0.12284	−0.12284	0.04858	0.02429	0.155307	20.58460272
L^2^ + VO(II)	−0.20163	−0.19237	−0.0093	0.00926	0.197	−0.197	0.00463	0.002315	4.191037	215.9827214
L^3^	−0.21252	−0.05654	−0.156	0.15598	0.13453	−0.13453	0.07799	0.038995	0.11603	12.82215669
L^3^ + VO(II)	−0.21881	−0.21008	−0.0087	0.00873	0.214445	−0.21445	0.004365	0.002183	5.267658	229.0950745
L^4^	−0.25291	−0.05654	−0.1964	0.19637	0.154725	−0.15473	0.098185	0.049093	0.121912	10.18485512
L^4^ + VO(II)	−0.20433	−0.19545	−0.0089	0.00888	0.19989	−0.19989	0.00444	0.00222	4.499551	225.2252252
L^5^	−0.17808	−0.0842	−0.0939	0.09388	0.13114	−0.13114	0.04694	0.02347	0.183188	21.30379207
L^5^ + VO(II)	−0.19719	−0.16607	−0.0311	0.03112	0.18163	−0.18163	0.01556	0.00778	1.060073	64.26735219

**Table 7:** Considerable bond lengths, charges, dipole moment (*D*), oscillator strength (*ʄ*), and excitation energies (*E*).

Compound	O^19^	N^11^	N^15^	N^16^	C^18^–O^19^	C^12^–N^11^	C^14^–N^15^	N^15^–N^16^	V^1^	V^2^	*D* (Debye)	*E* (nm)	*ʄ*
L^1^	−0.415473	−0.555221	−0.349927	−0.275635	1.224561	1.384229	1.287795	1.366391	—	—	5.1769	567.81	0.0316
L^1^ + VO(II)	−0.410181	−0.282766	−0.316313	−0.248429	—	—	—	—	0.933201	0.929331	11.5667	16513.7	0.003
L^2^	−0.410237	−0.555363	−0.348134	−0.275746	—	—	—	—	—	—	6.4963	576.26	0.0403
L^2^ + VO(II)	−0.420038	−0.397120	−0.322648	−0.250261	—	—	—	—	0.927552	0.915069	3.5504	31387.7	0.0008
L^3^	−0.416751	−0.044378	−0.050930	0.113237	1.317259	2.076019	1.772715	1.281712	—	—	5.3595	7613.39	0.002
L^3^ + VO(II)	−0.414856	−0.387699	−0.320321	−0.254947					0.955124	0.952544	16.6899	17266.3	0.0032
L^4^	−0.313357	−0.358945	−0.031267	−0.294059	1.223609	1.404653	1.286454	1.367672	—	—	4.4684	315.92	0.4055
L^4^ + VO(II)	−0.410181	−0.282766	−0.316313	−0.248429	—	—	—	—	0.933201	0.929331	11.5667	16513.7	0.003
L^5^	−0.354631	−0.480428	−0.209492	−0.312115	1.224058	1.384450	1.286411	1.367122	—	—	3.9661	600.17	0.043
L^5^ + VO(II)	−0.405024	−0.408894	−0.304735	−0.210573	—	—	—	—	0.905245	0.730601	8.9706	20988.6	0.002

**Table 8:** QSAR computation for optimized structures of perimidine compounds.

Function	L^1^	L^2^	L^3^	L^4^	L^5^
Surface area (approx.) (Å^2^)	425.73	488.36	496.79	464.04	465.53
Surface area (grid) (Å^2^)	623.02	661.15	661.91	644.29	642.82
Volume (Å^3^)	1060.57	1138.87	1134.04	1105.99	1105.72
Hydration energy (kcal/mol)	−8.29	−9.97	−17.83	−8.00	−8.02
Log P	2.53	1.53	−1.64	2.31	2.31
Reactivity (Å^3^)	132.87	139.25	138.92	137.59	137.59
Polarizability (Å^3^)	45.32	47.80	47.62	47.25	47.25
Mass (amu)	390.44	420.47	436.45	424.89	424.89

**Table 9:** Docking energy values (kcal/mol) of perimidine compounds (HL) and protein receptors complexes.

Ligands	p*K*a	Receptor	Est. free energy of binding	Est. inhibition constant (*K*_i_) (*µ*M)	vdW + bond + desolving energy	Electrostatic energy	Total intercooled energy	Frequency	Interacting surface
L^1^	10.96	4c3p	−7.92	1.57	−9.29	−0.06	−9.34	30%	859.778
		3bch	+355.37	—	+349.42	+0.06	+349.48	10%	665.36
		4zdr	−4.72	345.45	−5.97	−0.05	−6.02	20%	723.695
L^2^	10.96	4c3p	−7.75	2.09	−9.13	−0.13	−9.26	10%	983.377
		3bch	+490.76	—	+473.39	+0.00	+473.39	10%	662.71
		4zdr	−4.32	686.85	−5.97	−0.02	−5.99	20%	758.018
L^3^	10.95	4c3p	+647.56	—	+644.74	+0.01	+644.75	10%	718.318
		3bch	+709.10	—	+699.61	−0.05	+699.56	10%	661.43
		4zdr	−4.66	385.21	−6.46	+0.07	−6.39	10%	621.389
L^4^	10.96	4c3p	−7.28	4.62	−8.57	−0.03	−8.60	20%	929.747
		3bch	+552.25	—	+549.59	−0.03	+549.56	30%	710.605
		4zdr	−4.67	376.04	−6.13	−0.19	−6.32	20%	595.541
L^5^	10.95	4c3p	−8.41	683.74	−9.81	−0.00	−9.81	20%	925.161
		3bch	+663.87	—	+661.94	+0.01	+661.95	10%	703.598
		4zdr	−4.84	284.49	−6.39	+0.04	−6.35	10%	690.838

**Table 10:** IC_50_ of some tested compounds against liver (HepG2), breast (MCF-7), and colon (HCT116) cancer cell lines.

Cell type	IC_50_ (*µ*g/ml)										
	L^1^	VO(II)-L^1^	L^2^	VO(II)-L^2^	L^3^	VO(II)-L^3^	L^4^	VO(II)-L^4^	L^5^	VO(II)-L^5^	Doxorubicin
MCF-7	19.68	23.06	25.92	93.92	15.50	>100	24.96	3.42	11.44	>100	0.60
HepG2	19.79	19.94	27.23	55.67	11.01	>100	28.25	1.27	9.91	>100	0.34
HCT116	19.15	22.93	13.27	95.17	15.53	>100	26.24	1.66	23.30	>100	0.39

## Data Availability

The data used to support the findings of this study are available from the corresponding author upon request.

## Supplementary Materials

Supplementary Materials Figure S1: ^1^H·NMR spectra of L^4^ and L^5^ perimidine ligands. Figure S2: mass spectra of L^3^ and L^4^ ligands. Figure S3: X-ray patterns of four perimidine ligands. Figure S4: SEM images of perimidine ligands and their VO(II) complexes. Figure S5: interacting protein-inhibitor complexes for L^1^, L^2^, L^3^, L^4^, and L^5^ with 4c3p (1) and 3bch (2) receptors (A–E, respectively). Figure S6: interacting hp plot: LH, LOMe, LNO_2_, LClP, and LClM with 4c3p(1) and 3bch(2) receptors (A–E, respectively). Figure S7: 2D plot forms: L^1^, L^2^, L^3^, L^4^, and L^5^ with 4c3p (1), 3bch (2), and 4zdr (3) receptors (A–E, respectively). Figure S8: dose response curves of perimidine-VO(II) complexes against MCF-7, HCT116, and HepG2 cancer cells.
