# Supplementary material for: Synthesis of Novel VO(II)-Perimidine Complexes: Spectral, Computational, and Antitumor Studies
Source: Bioinorg Chem Appl. 2018 Sep 6;2018:7176040. doi: 10.1155/2018/7176040 (PMC6151213; doi:10.1155/2018/7176040)
Supplement: Supplementary Materials — Figure S1: 1H·NMR spectra of L4 and L5 perimidine ligands. Figure S2: mass spectra of L3 and L4 ligands. Figure S3: X-ray patterns of four perimidine ligands. Figure S4: SEM images of perimidine ligands and their VO(II) complexes. Figure S5: interacting protein-inhibitor complexes for L1, L2, L3, L4, and L5 with 4c3p (1) and 3bch (2) receptors (A–E, respectively). Figure S6: interacting hp plot: LH, LOMe, LNO2, LClP, and LClM with 4c3p(1) and 3bch(2) receptors (A–E, respectively). Figure S7: 2D plot forms: L1, L2, L3, L4, and L5 with 4c3p (1), 3bch (2), and 4zdr (3) receptors (A–E, respectively). Figure S8: dose response curves of perimidine-VO(II) complexes against MCF-7, HCT116, and HepG2 cancer cells. [file 7176040.f1.pdf]

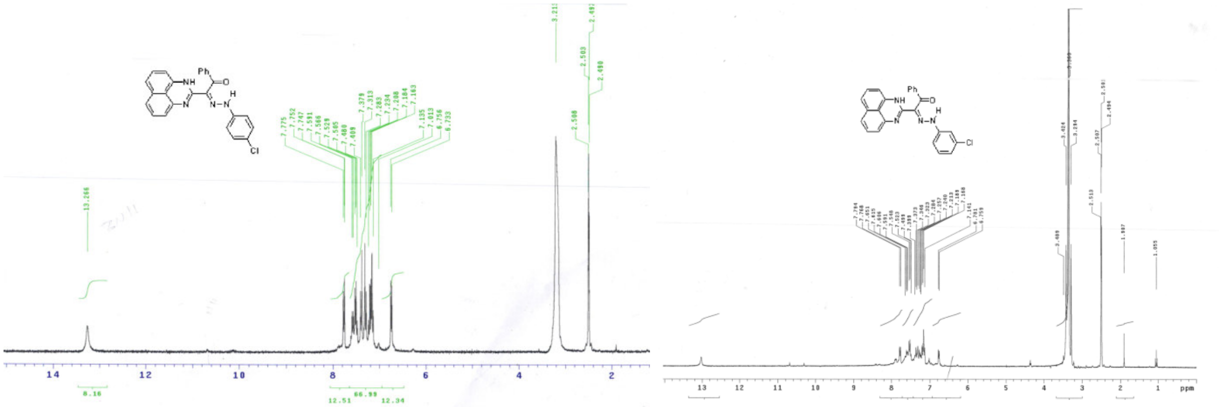

Fig. 1S . ^{1}HNMR spectra of LClP and LClM perimidine ligands

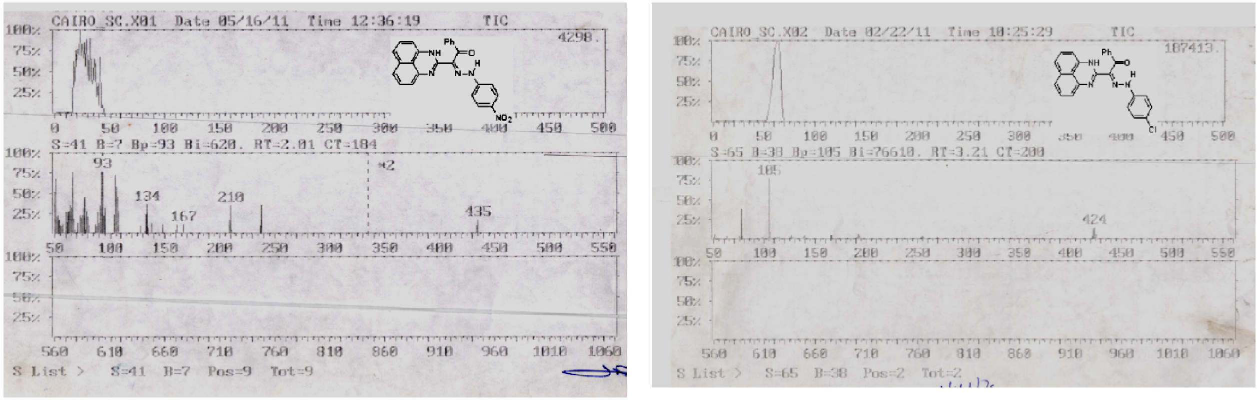

Fig. 2S. Mass spectra of LNO_2 and LClP ligands

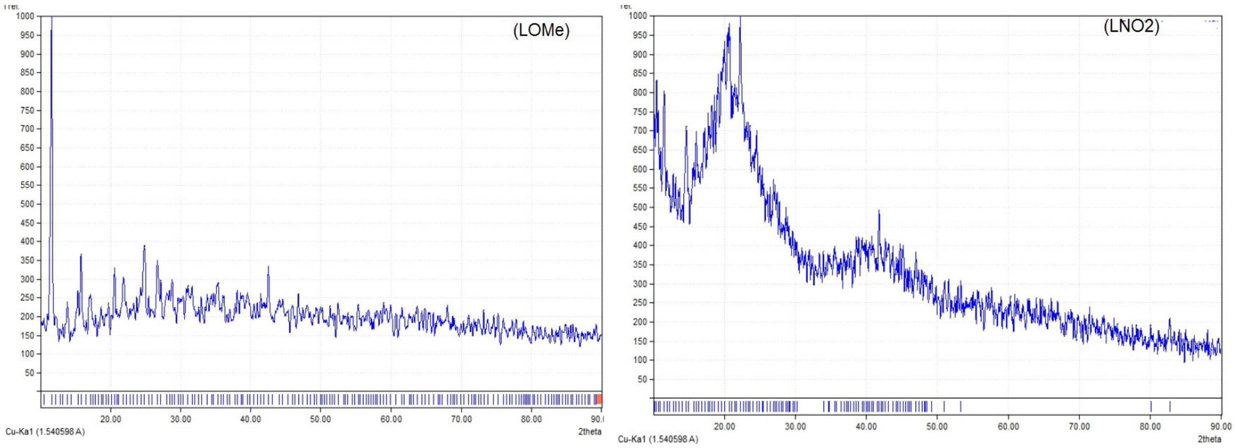

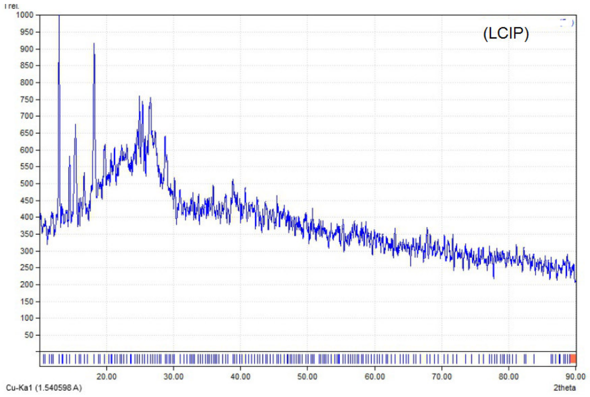

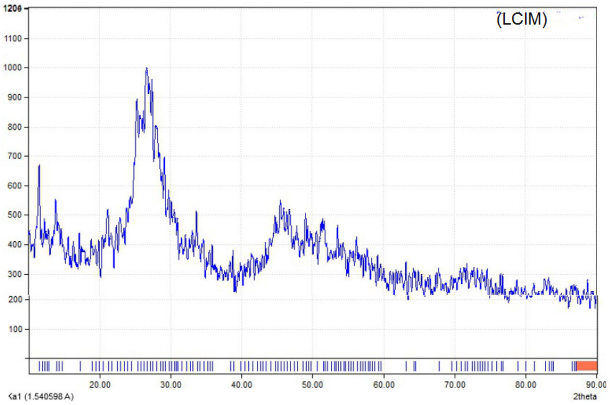

Fig. 3 S. X-Ray patterns for four perimidine ligands

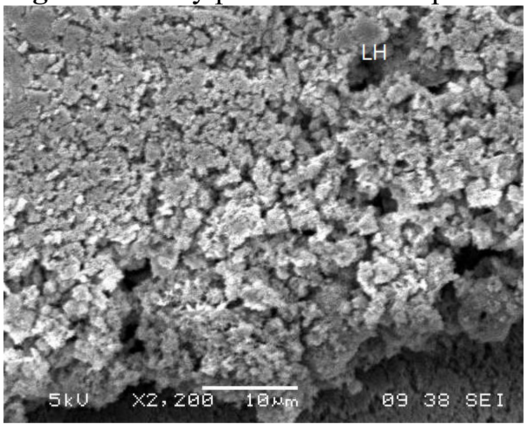

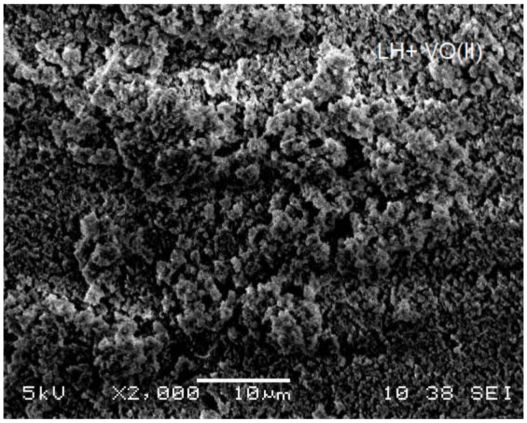

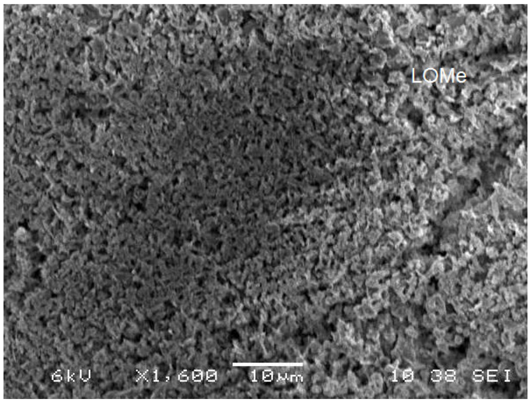

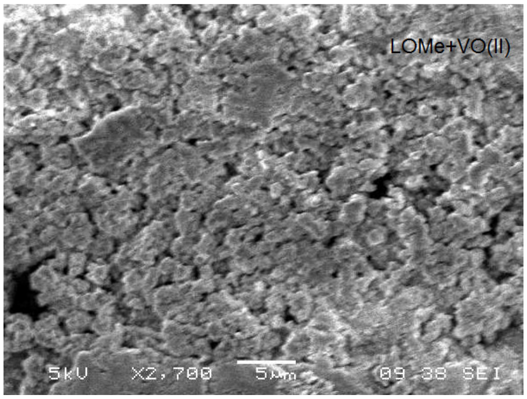

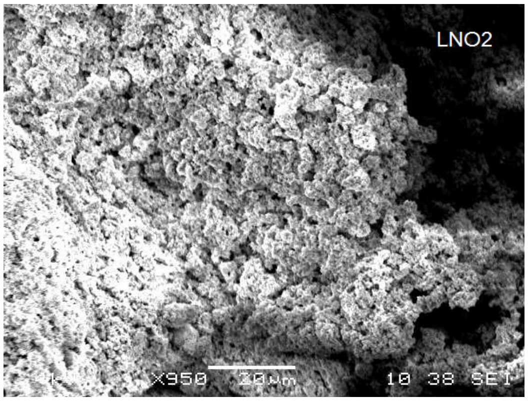

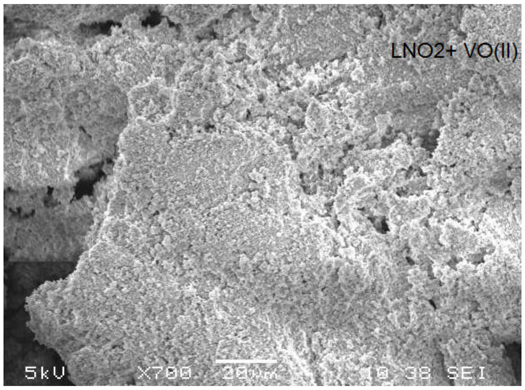

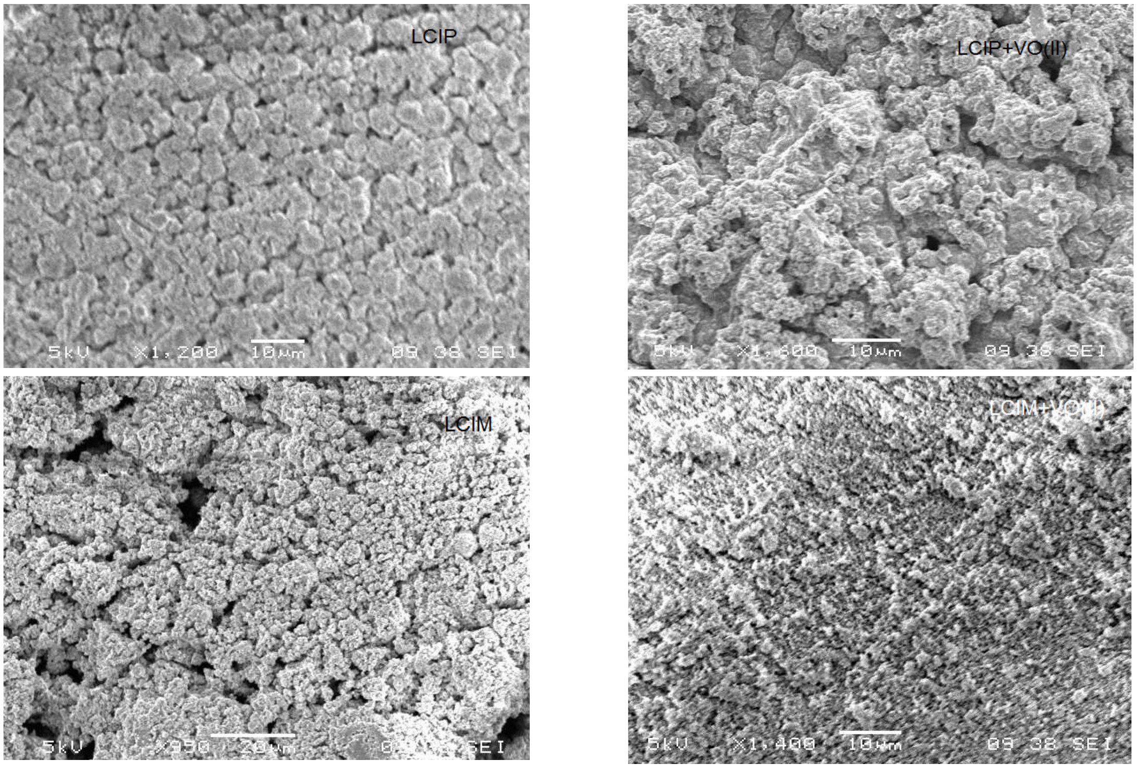

Fig. 4S . SEM images of perimidine ligands and their VO(II) complexes

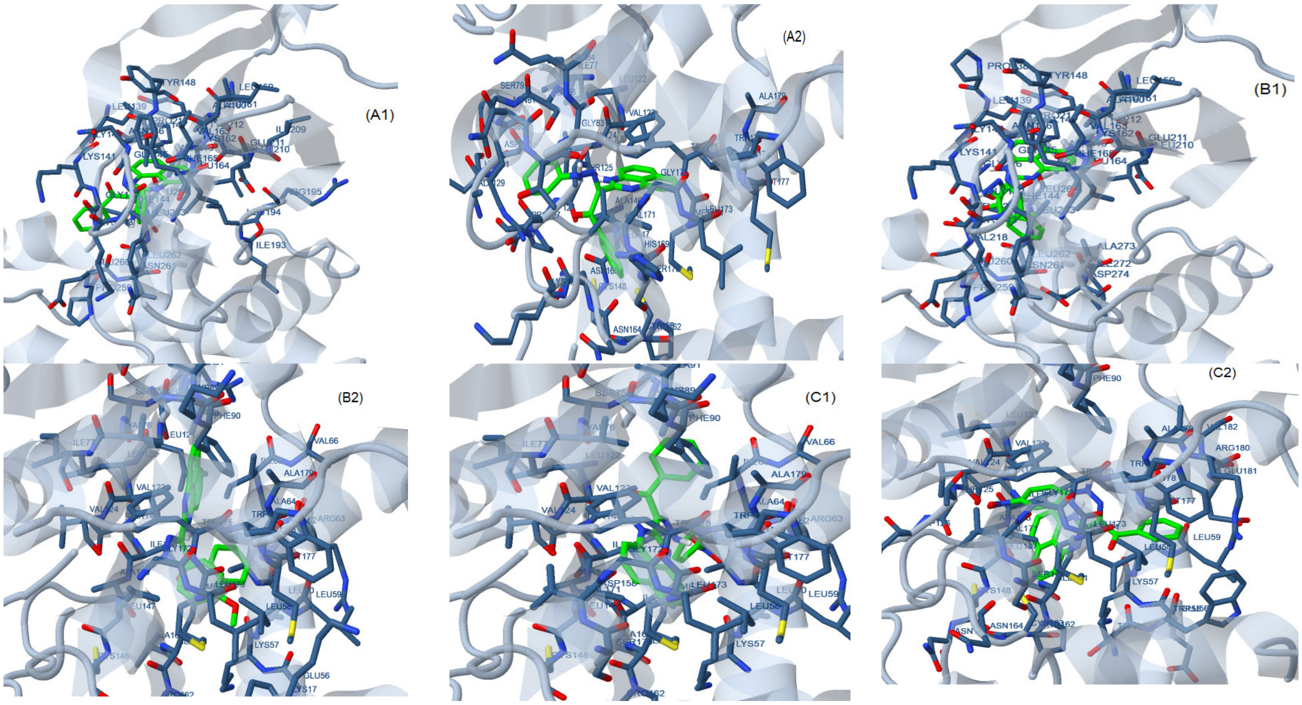

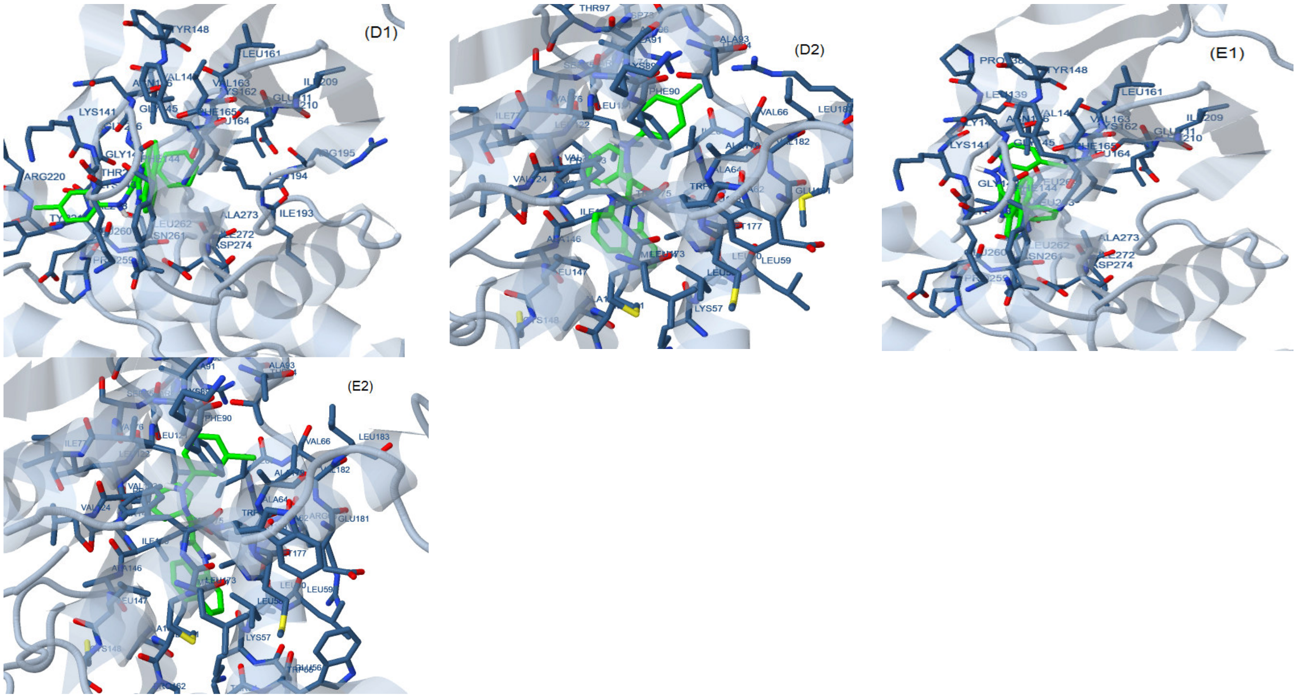

Fig. 5S . Interacting Protein- inhibitor complexes for ; LH, LOMe, LNO_2, LClP and LClM with 4c3p (1) and 3bch (2) receptors (A-E, respectively)

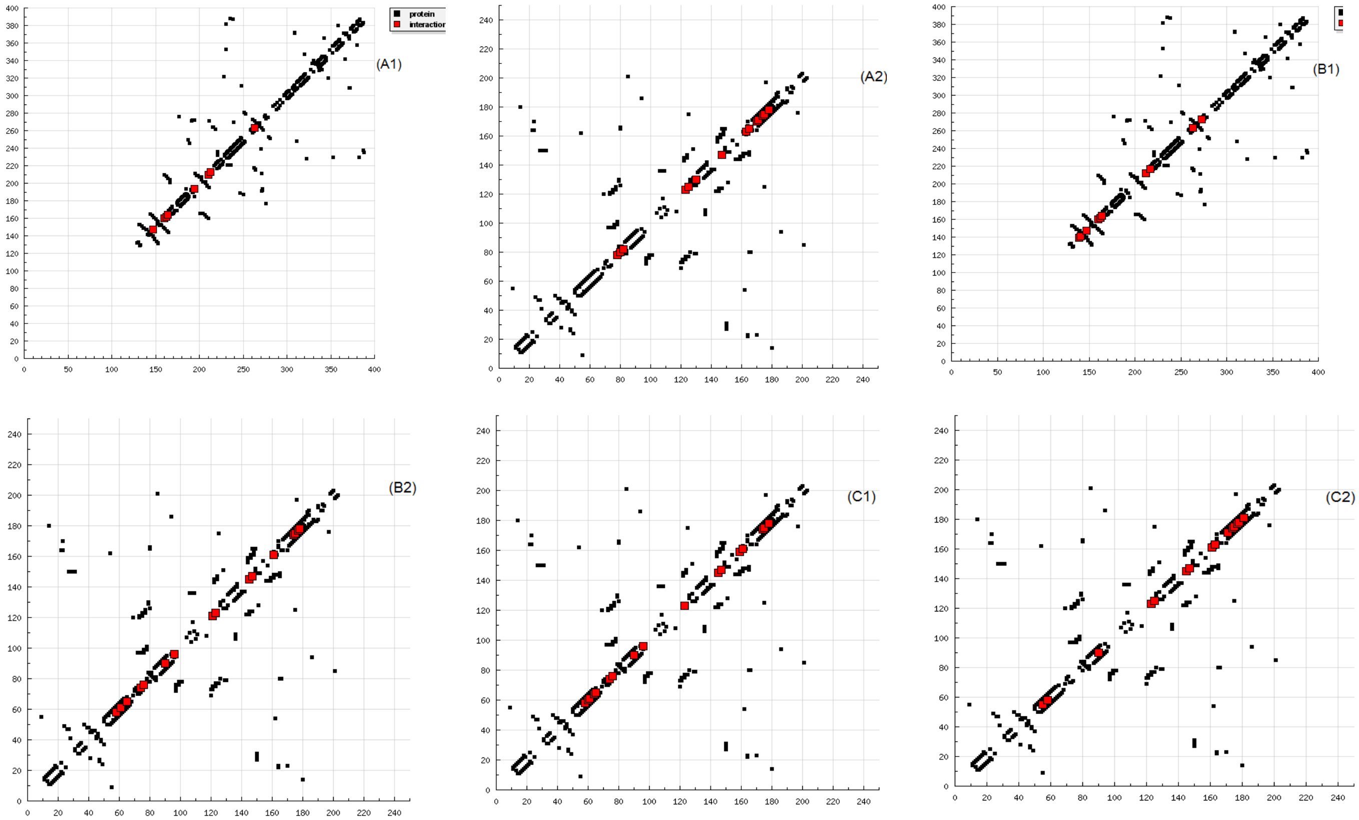

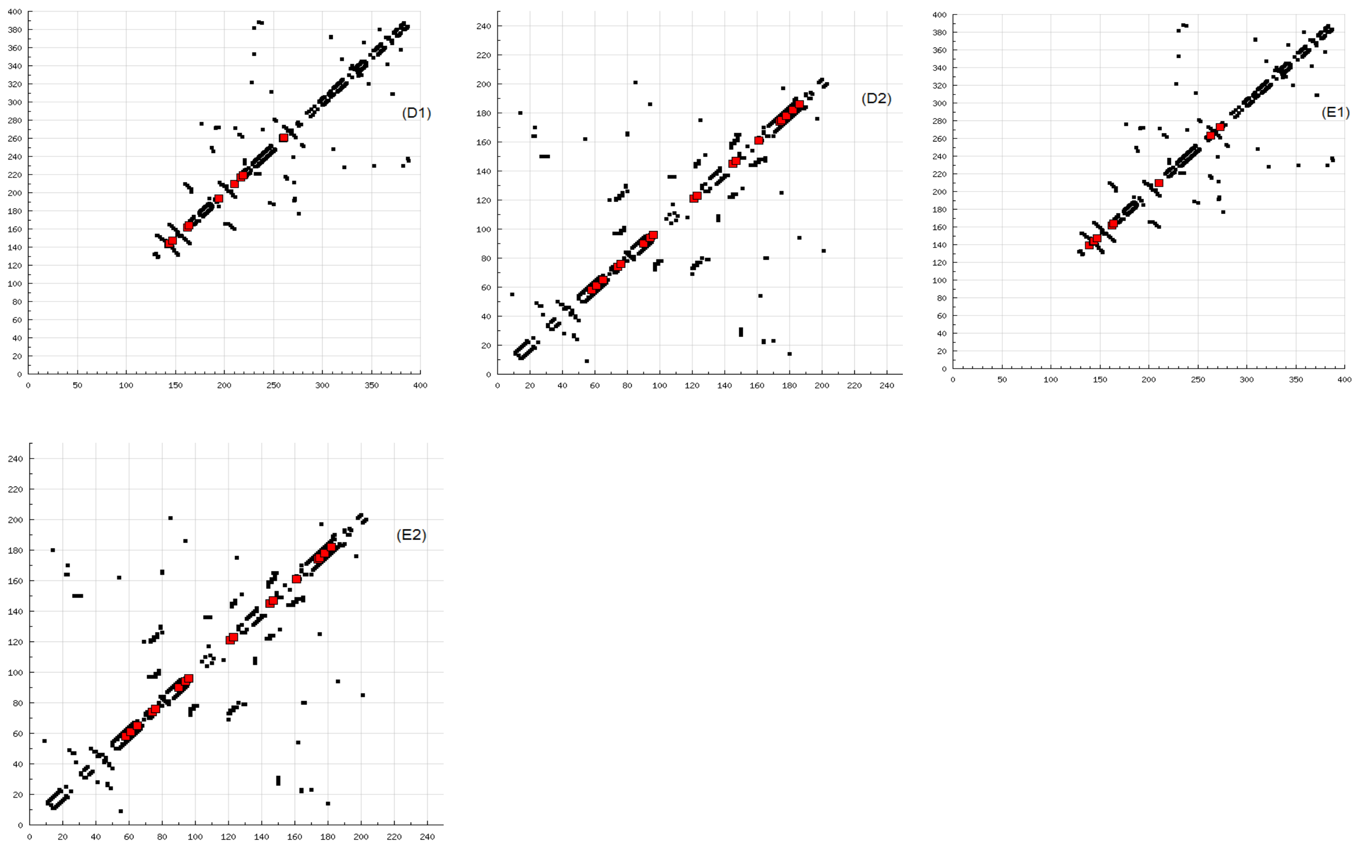

Fig.**6S** . Interacting hp plot ; LH, LOMe,LNO_2,LClPand LClM with 4c3p(1) and 3bch(2) receptors(A-E, respectively)

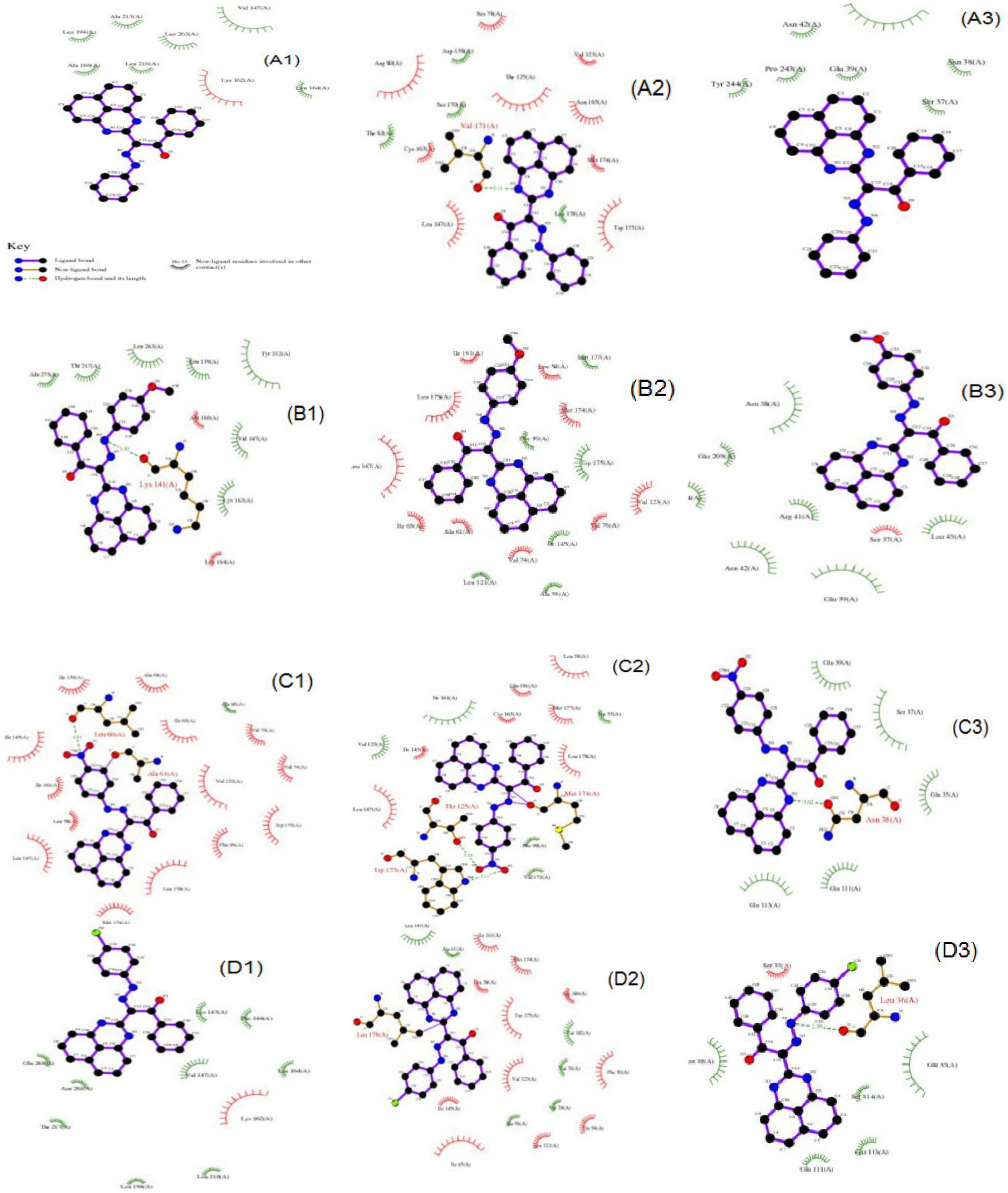
(A1)
(A2)
(A3)
Key
Ligand bond
Non-ligand bond
Hydrogen bond and its length
Non-ligand residues involved in other contact(s)
(B1)
(B2)
(B3)
(C1)
(C2)
(C3)
(D1)
(D2)
(D3)

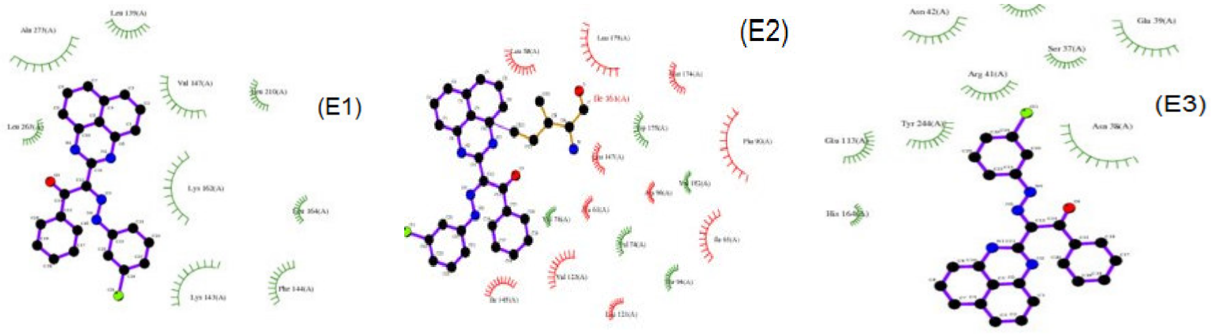

Fig. 7S. 2D plot forms ; LH, LOMe, LNO_2, LClP and LClM with 4c3p (1), 3bch (2) and 4zdr (3) receptors (A-E, respectively)

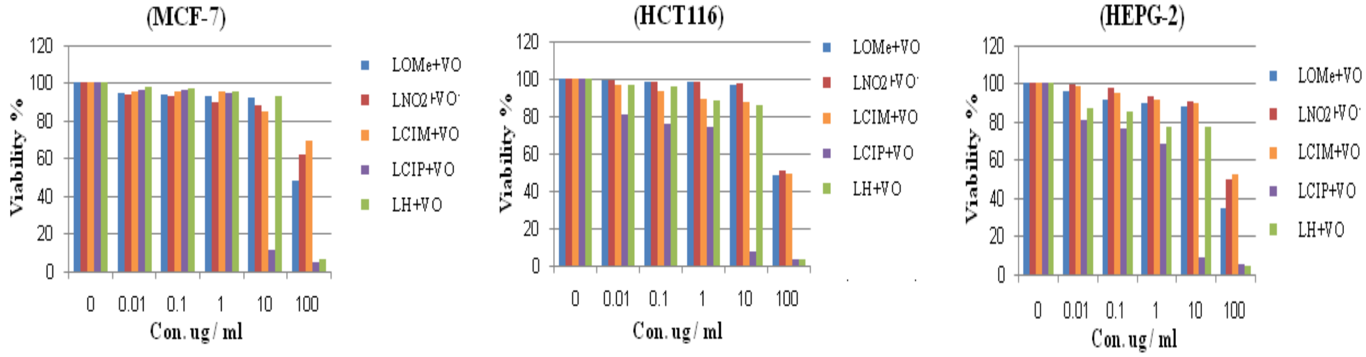

Fig.8S . Dose response curves of perimidine-VO(II) complexes against MCF-7, HCT-116 and HEPG-2 cancer cells
